# Supplementary material for: Treatment outcomes of cutaneous leishmaniasis due to Leishmania aethiopica: A systematic review and meta-analysis
Source: PLoS One. 2023 Nov 2;18(11):e0293529. doi: 10.1371/journal.pone.0293529 (PMC10621858; doi:10.1371/journal.pone.0293529)
Supplement: S1 Table — (DOCX) [file pone.0293529.s001.docx]

**S1 Table**. **Quality of studies included in the systematic review and meta-analysis.**

**Table A**. The quality of non-randomized studies using the NOS scale.

| Author/year | Study design | Treatment group representative | Control group representative | Treatment details given | Outcome not pre-existing | Comparability of the group | Independent outcome ascertainment | Follow-up>=3months | Loss to follow-up<10% | Total score |
| --- | --- | --- | --- | --- | --- | --- | --- | --- | --- | --- |
| Bryceson et al. /1970 [[26](#_ENREF_26)] | Case series | 0 | 0 | * | * | 0 | 0 | 0 | 0 | 2 |
| Belhu et al. /1978 [[28](#_ENREF_28)] | Case series | 0 | 0 | * | * | 0 | 0 | 0 | 0 | 2 |
| Chulay et al. /1983 [[8](#_ENREF_8)] | Case series | 0 | 0 | * | * | 0 | 0 | 0 | 0 | 2 |
| Padoverse et al. /2009 [[14](#_ENREF_14)] | Cohort | * | * | * | * | 0 | 0 | * | 0 | **5** |
| Negera et al. /2012 [[15](#_ENREF_15)] | Cohort | * | * | * | * | 0 | 0 | * | 0 | **5** |
| Fikrie et al. /2016 [[16](#_ENREF_16)] | Retrospective evaluation | * | * | * | * | 0 | * | 0 | 0 | **5** |
| Seife et al. /2018 [[35](#_ENREF_35)] | Cohort | * | * | * | * | 0 | * | * | * | **7** |
| Tegegne et al. /2020 [[36](#_ENREF_36)] | Case report | 0 | 0 | * | * | 0 | 0 | * | * | 4 |
| van Henten et al. /2021 [[17](#_ENREF_17)] | Cohort | * | * | * | * | 0 | * | * | * | **7** |
| Berhe et al. /1995 [[34](#_ENREF_34)] | Case report | 0 | 0 | 0 | * | 0 | 0 | * | * | 3 |
| Barnetson, et al. /1978 [[27](#_ENREF_27)] | Case series | 0 | 0 | 0 | * | 0 | 0 | 0 | * | 2 |
| Zaar et al. /1983 [[30](#_ENREF_30)] | Case series | 0 | 0 | * | * | 0 | 0 | 0 | * | 3 |
| Henriksen et al. /1983 [[31](#_ENREF_31)] | Case series | 0 | 0 | * | * | 0 | 0 | 0 | * | 3 |
| Teklemariam et al. /1994 [[33](#_ENREF_33)] | Case series | 0 | 0 | * | * | 0 | 0 | * | * | 4 |
| Tesfa et al. /2022 [[37](#_ENREF_37)] | Retrospective evaluation | * | * | * | * | 0 | 0 | 0 | * | **5** |
| Weinrauch et al. /1987 [[9](#_ENREF_9)] | Case series | 0 | 0 | 0 | * | * | 0 | 0 | * | 3 |
| Mengeot et al. /2022 [[11](#_ENREF_11)] | Case report | 0 | 0 | 0 | * | * | 0 | * | * | 4 |
| Zanger et al. /2011 [[10](#_ENREF_10)] | Case report | 0 | 0 | 0 | * | * | * | 0 | 0 | 3 |
| Tilahun et al. /2022 [[19](#_ENREF_19)] | Cohort | * | * | * | * | * | 0 | * | * | **7** |

**Table B**. The risk of bias assessment for randomized studies.

| Author/year | Experimental | Comparator | Outcome | Randomization process | Deviations from the intended interventions | Missing outcome data | Measurement of the outcome | Selection of the reported result | Overall | Key |
| --- | --- | --- | --- | --- | --- | --- | --- | --- | --- | --- |
| Van der Meulen et al. /1981 [[29](#_ENREF_29)] | Rifampicin and Isoniazid | Meglumine antimonate | Cure, improvement |  |  |  |  |  |  | Low risk |
| Akuffo et al. /1990 [[32](#_ENREF_32)] | Itraconazole | Placebo | Cure, failure, partial improvement |  |  |  |  |  |  | Some concerns |
| Na-Bangchang et al. /2016 [[13](#_ENREF_13)] | Shuinko ointment | Placebo | cure, partial response, and failure |  |  |  |  |  |  | High risk |
