# Supplementary material for: Treatment outcomes of cutaneous leishmaniasis due to Leishmania aethiopica: A systematic review and meta-analysis
Source: PLoS One. 2023 Nov 2;18(11):e0293529. doi: 10.1371/journal.pone.0293529 (PMC10621858; doi:10.1371/journal.pone.0293529)
Supplement: S2 Table — (DOCX) [file pone.0293529.s002.docx]

**S2 Table. Characteristics of studies and treatment outcomes.**

**Table A**. Treatment outcomes of CL due to *L. aethiopica* in case report and case series studies.

| Author/year | Study design | Diagnosis confirmation method | Sample size | Clinical phenotype of CL | Treatment detail (route, dose, dosage) | Characteristics of participants | Treatment outcomes |
| --- | --- | --- | --- | --- | --- | --- | --- |
| Bryceson A. et al. /1970 [[26](#_ENREF_26)] | Case series | Photograph, smears, biopsy and leishmanin skin testing | 33 | DCL | Pentamidine IM 4mg/kg daily, weekly, fortnightly, monthly; Pentostam 10-20mg/kg IM/IV daily 5-30 days; Combined SSG and pentamidine; Glucantime 3mk/kg/day IM for 10-21 days; Astiban 10mg/kg/day IM twice weekly 5-7 injections; Chloroquine 150 mg, one or two tablet PO three times per day for 10 days-2 months daily; Paludrine 100 mg PO one tablet three times daily for 10, 20 and 30 days; Primaquine 15 mg PO one or two tablets daily for 4-8 weeks; 349-C59 15 mg one to four tablets PO daily for 10-30 days; Cycloguanil pamoate IM 3-10 weeks; Griseofulvin 250 mg PO one tablet daily for 10-30 days; Amphotericin B 1 mg/kg IV alternate days up to 4 months; Macrocyclon IM 2-3 times/week, 4-8 weeks; Surgical excision of DCL lesion | The age of participants was as low as 8 years, but the highest age was not recorded except mentioning 33 years of age  The highest age was not reported  Participants’ gender was not recorded except for all, except a girl and a man  All from Ethiopia | **Cure:** Pentamidine (n=7); Chloroquine (n=3); Primaquine (n=1); Amphotericin B (n=1); Surgery (n=1);  **Toxicity:** Pentamidine (3+); Pentostam (+); Glucantime (+); Astiban (+); Chloroquine (+); Amphotericin B (3+)  **Unresponsive:** Pentamidine (n=24); Pentostam (n=17); Glucantime (n=8); Astiban (n=7); Chloroquine (n=12); Primaquine (n=6); 349-C59 (n=5); Paludrine (n=3); Cycloguanil pamoate (n=2); Griseofulvin (n=7); Amphotericin B (n=3); Macrocyclon (n=4); Surgery (n=1)  The outcome was measured clinically, with smears, biopsy and leishmanin skin testing |
| Belhu et al. /1978 [[28](#_ENREF_28)] | Case series | Histology | 5 | MCL | 500 mg Metronidazole three times daily PO for four to eight weeks | 2 females and 3 males  Age not reported  All from Ethiopia | No change in lesions in all patients, two patients reported a burning sensation |
| Chuluy et al. /1983 [[8](#_ENREF_8)] | Case series | Smear and culture | 3 | Not specified | High dose of IV-SSG twice daily for 30 days | Male  Age range 18-23 years  Kenyan | Good response, the disappearance of parasite from culture and smear in 14-27 days, 3-18 months of follow-up; Minor side effects |
| Zanger et al. /2011 [[10](#_ENREF_10)] | Case report | Histology and culture | 1 | Not specified | IV Liposomal Amphotericin B 200mg/day for 22 days | A 38-year-old male **immigrant** from Eritrea to Germany | The lesion showed improvement without recurrence at 12 months of follow-up  Improvement: Clinical at 12 months |
| Tegegne et al. /2020 [[36](#_ENREF_36)] | Case report | Smear | 1 | MCL progressed to DCL | **Combination**: sodium stibogluconate (20 mg/kg/day IM for 30 days) and paromomycin (15 mg/kg/day IM for 30 days) combination therapy, then prolonged treatment with SSG (20 mg/kg/day IM for 60 days) | Male  38 years old  An Ethiopian | Non-responsive |
| Berhe et al. /1995 [[34](#_ENREF_34)] | Case series | Smear | 2 | MCL and reactivated LCL | 20 mg/kg/day IV-SSG for 30 days | All male  Age 29 and 38 years  Both HIV positive | Recurrence at five months of follow-up |
| Barnetson et al. /1978 [[27](#_ENREF_27)] | Case series | Smear and biopsy | 8 | MCL and DCL | Pentamidine (7), Cycloguanil pamoate (1), route of drug administration not stated  **Combination**: pentamidine and cycloguanil pamoate | Six male and two female  Age range of males 15-30 years  Female 12 years and 18 years  All cases had concomitant leprosy  All from Ethiopia | Six respond well to pentamidine, two were resistant to antileishmanial drugs (one patient was treated with pentamidine and cycloguanil pamoate) |
| Zaar et al. /1983 [[30](#_ENREF_30)] | Case series | Biopsy | 2 | DCL | Pentamidine 4mg/kg/IM daily for two weeks | Female, age 28 years  Male, age 35 years  Both from Ethiopia | Parasite number decrease  Smear microscopy and biopsy |
| Weinrauch et al. /1987 [[9](#_ENREF_9)] | Case series | Smear | 3 | Not specified | **Combination**: 15% paromomycin sulfate and methyl 12% benzethonium chloride ointment for 10 days  One case was treated for 40 days; one for 20 days; and one for 10 days  Application of ointment extended for 10 or 20 days when improvement is not observed | Six and ten years ole girls, and 26 years old man  Immigrants from Ethiopia to Israel | Clinical improvement and complete healing without scar formation after 40 days at four months  Smear and culture negative after 20 days;  Parasite eradication after 10 days |
| Henriksen et al. /1983 [[31](#_ENREF_31)] | Case series | Smear and histology | 3 | DCL | **Combination**: 1g 25% Chlorpromazine and 25% 50g Vaseline for one month  Topical application | Age and sex not specified  All from Ethiopia | Clinical signs of inflammation disappeared in one and lesion size decreased in one |
| Teklemariam et al. /1994 [[33](#_ENREF_33)] | Case series | Smear and culture | 3 | DCL | Two cases were treated with Aminosidine IM 14 mg/kg/day for 60 days; one case was treated with a **combination** of the same dose of Aminosidine and 10 mg/kg/day SSG IM daily.  All were treated for two months after the parasitological cure | Two males and one female  Age not recorded  Both Ethiopian | Two male and one female case relapsed after 60 days of Amnosidne and one non-relapsed case then cure with minimal side-effects  Follow-up two to 21 months |
| Mengeot. et al. /2022 [[11](#_ENREF_11)] | case report | Histology and PCR | 1 | LCL | Intravenous amphotericin B liposomal for five days; the second course started but discontinued after 4 days due to toxicity, then a **combination** of oral posaconazole plus phototherapy;  A daily 1.5 g/5 ml intramuscular injection of meglumine antimoniate for 45 days | A 64 years old male  Belgian  Travelled from Ethiopia to Belgium | Relapsed after five days of amphotericin B liposomal; no response;  Complete resolution of the lesion after meglumine antimonate  Positive outcome: Complete resolution of the lesion at 45 days and no relapse at six months  Cure not declared |

Note: Note: PCR: Polymerase chain reaction; LCL: Localized cutaneous leishmaniasis; MCL: Mucocutaneous leishmaniasis; DCL: Diffuse cutaneous leishmaniasis; PLWH: People live with HIV; IM: Intramuscular; IV: intravenous; SSG: Sodium stibogluconate

**Table B**. Treatment outcomes of CL due to *L. aethiopica* in cohort and retrospective chart review studies.

| Author/year | Study design | Diagnosis confirmation method | Sample size (N) | Clinical phenotype of CL | Treatment detail (route, dose, dosage) | Characteristics of participants | Treatment outcomes |
| --- | --- | --- | --- | --- | --- | --- | --- |
| Padovese et al. /2009 [[14](#_ENREF_14)] | Cohort | Smear, biopsy and clinical | 154 | LCL, MCL and DCL | **Combination** of route IL and IM-Meglumine antimonate (MA) 20mg/kg/day IL 1 ml every 3 days for 4 weeks;  For resistant cases and relapse cases: Pentamidine 4mg/kg IM every second day for 20 days | Majority of the participants were school children and were males  Most participants were between age five and 44  Two participants were < 5 years, and 12 were > 45 years  Five cases were HIV positive  Duration of follow-up 180 days | About 69% had a positive outcome (cure): 21% with IL-MA; 47% with IL-MA and IM-MA.  Seven/eight cases resistant to MA who were treated with pentamidine for 20 days had a cure after six months  Twenty-three (15%) had negative outcomes (unspecified), relapse (14%), and resistance to meglumine antimonate (28%); four cases discontinue treatment because of serious side effects.  52% of relapsed cases were re-treated with IV-MA, but not cured  Monthly follow-up for six months  Did not report outcome across the clinical phenotypes of CL  **Cure**: Free from clinical and negative microscopic evidence of disease at 6 months |
| Negera et al. /2012 [[15](#_ENREF_15)] | Cohort | Smear, culture, histology and PCR | 109 | Not differentiated | Cryotherapy used three to four sessions per lesion per visit applied for 10-30 seconds every 20-second interval  IM-SSG 20 mg/kg daily for 30 days | All ages and sex  In the cryotherapy group, 28.1% age below 10 years  Among those who treated with SSG 70% were 11-20 years  Duration of follow-up 90 days | About 81% and 85% of patients treated with cryotherapy and SSG were cured, respectively. Among cases treated with cryotherapy, 14% were dropouts, and 6% were unresponsive.  Non-responsive case to cryotherapy cured after IM SSG 20mg/kg/day for 30 days. Two unresponsive cases to SSG were treated with SSG + Cryotherapy, and one case dropped the SSG treatment.  Follow-up at one, three and six months after completion of treatment  **Cure**: Clinical cure at three months |
| Fikrie et al. /2016 [[16](#_ENREF_16)] | Retrospective chart review | Smear and clinical | 121 | LCL, MCL and DCL | 126 (61 LCL, 58 MCL, 7DCL) cases treated IM/IV-SSG 20mg/kg/day daily for 30 days. One LCL case was treated with Cryotherapy, and one MCL with AmBisome  58 MCL cases treated with SSG and one with Cryotherapy  Six cases treated with IM-SSG 20 mg/kg/day for 30 days and one case for 3.5 months | Both sex  Median age 23 years  Majority of participants were male  Nearly 30% of participants were below 18 years of age  28.6% of participants took traditional treatment  Outcome measured based on the 28^th^ day result  Five cases were HIV positive | Only 13.5% of cases treated with SSG were cured after 30 days of treatment. About 38.3% required treatment extension.  About 32% of cases treated with SSG required treatment extension beyond one month due to unresponsiveness eight cases or partial response 32 cases.  Five DCL cases showed partial response and one was unresponsive  **Cure**: Clinical cure at one month |
| Seife et al. /2018 [[35](#_ENREF_35)] | Cohort | Smear, biopsy and clinical | 97 | LCL, MCL and DCL | **Combination** and monotherapy: 26 LCL cases treated with Cryotherapy, and 26 treated with Cryotherapy + IL SSG  14 MCL cases treated with SSG IM+ Allopurinol and 14 cases treated with SSG IM +IL  10 DCL cases treated with SSG IM+ Allopurinol and 7 treated with SSG+ Cryotherapy+ IL-SSG | Both sex  Age range 16-45 years  Majority of the participants were male  33% had applied herbal treatment before their visit to hospital  Six cases had comorbidity  Duration of follow-up 90 days | Overall, 94.2% of LCL cases were cured (92% with Cryotherapy and 96% with Cryotherapy + IL SSG), 82.2% of MCL cases were cured (79% SSG IM + Allopurinol, and 86% with SSG IM +IL-SSG), 83% of DCL cured (80% SSG IM + Allopurinol, and 86% with SSG + Cryotherapy + IL-SSG)  **Cure**: Clinical cure at one or three months after treatment complete therapy |
| van Henten et al. /2021 [[17](#_ENREF_17)] | Cohort | Smear, PCR (five cases) and clinical | 58 | LCL, MCL, DCL and LR | Miltefosine daily for 28 days (>45 kg 150mg/day, 30-44 kg 100mg/day and allometric dose for children below 30kg). Patients at Gondar were treated as an outpatient, but cases were admitted for one week in Boru | 2/3^rd^ of the study participants were male  Median age 21 years old  Most lesions seen on the face  Three HIV positive cases  Duration of follow-up 180 days  74.3% used prior traditional treatment | About 48.7% of patients reached cure at day 180, and 32.3% relapsed  **Cure**: Clinical cure at one, three and six months |
| Tesfa. et al. /2022 [[37](#_ENREF_37)] | Retrospective chart review |  | 66 | LCL, MCL and DCL | **Combination** and monotherapy: 55 with IM/IV SSG/MA; 6 cases treated SSG + Paromomycin; 2 Amphotericin B; 3 Dapsone; 17 SSG IL; 2  Cryotherapy; 11 cases had treatment extension | Both sex  Age range 1-60 years  The majority were below 25 years of age  The majority were male  Most lesions observed on the face  Outcome recorded at 90 days used | Overall, all 61% showed cure, 16.7% partial improvement  At three months follow-up  55% cure for IM-SSG; intralesional antimonial showed a cure rate of 94%; non who take dapsone showed improvement, and only the one who got an extension was cured  Cure: Clinical cure at three months |
| Tilahun et al. /2022 [[19](#_ENREF_19)] | Cohort study | Smear, clinical and biopsy | 72 | LCL | IL-SSG weekly for a maximum of six doses  Duration of follow-up 90 days | Both sex  Median age 20 years  More than half male  Majority of the lesions stayed more than 6 months  Body parts affected were mainly the face and extremities  73.5% use traditional treatment prior | Overall cure rate 60%  Worsening 5.9%  No response 2.8%  Partial response 16.9%  13.9% good response |

Note: Note: PCR: Polymerase chain reaction; LCL: IL: Intralesional; Localized cutaneous leishmaniasis; MCL: Mucocutaneous leishmaniasis; DCL: Diffuse cutaneous leishmaniasis; IM: Intramuscular; IV: intravenous; SSG: Sodium stibogluconate; LR: Leishmania recidivans

**Table C**. Treatment outcomes of CL due to *L. aethiopica* in clinical trial studies.

| Author/year | Diagnosis | Sample size | Clinical phenotype of CL | Treatment detail (route, dose, dosage) | Patient characteristics | Treatment outcomes among intervention group | Treatment outcomes among the control group |
| --- | --- | --- | --- | --- | --- | --- | --- |
| Akufo et al. /1990 [[33](file:///C:\Users\user\Desktop\PhD%20seminars\Systematic%20review\Search\SRM%20manuscript\Comments%20from%20supervisors\Revised\plosonedecisionrevisionrequiredponed2303546em\Second%20round%20review\Supporting%20file\S2_Table.doc.docx#_ENREF_33)] | Smear | 14 | LCL and DCL | Itraconazole 50 mg per day for 4 weeks | A total of 14 patients participated, all 4 DCL patients were male and ten were LCL patients; three females were in the itraconazole group and one female in the placebo group  Duration of disease ranges from one to 17 years for DCL and 2 months to 2 years for LCL | Clinically, two of four LCL treated improved after 4 weeks. Four of five LCL showed a reduction in the number of parasites from culture and smear.  Five of seven reported improvements after 4 weeks.  Clinically, after 4 weeks all taking itraconazole had active lesions.  Follow-up after one month. No toxicity was reported from all treated with itraconazole.  Itraconazole was not effective for CL | Four of the 5 placebo-treated LCL patients clinically improved; five of seven reported improvements after 4 weeks; three of five showed a reduction in the number of parasites from culture and smear. |
| Na-Bangchang et al. /2016 [[9](file:///C:\Users\user\Desktop\PhD%20seminars\Systematic%20review\Search\SRM%20manuscript\Comments%20from%20supervisors\Revised\plosonedecisionrevisionrequiredponed2303546em\Second%20round%20review\Supporting%20file\S2_Table.doc.docx#_ENREF_9)] | Smear and clinical | 40 | LCL | Shuinko ointment twice daily for four weeks | 20 patients each in Shuinko and Placebo group  Shuinko applied twice a day for four weeks | At 16 weeks follow-up, the Shuinko group showed a 69% reduction in lesion size versus 22% in placebo with a fast reduction of lesion size in the Shuinko group. In the Shuinko group lesion size reduced to 31% versus 78% in the placebo group  Five cured and six partial responses, and eight failure  Shuinko ointment recommended as adjuvant treatment for LCL | 4 cured and 6 partial response |
| Van der Meulen et al. /1981 [[29](file:///C:\Users\user\Desktop\PhD%20seminars\Systematic%20review\Search\SRM%20manuscript\Comments%20from%20supervisors\Revised\plosonedecisionrevisionrequiredponed2303546em\Second%20round%20review\Supporting%20file\S2_Table.doc.docx#_ENREF_29)] | Smear and leishmanin skin test | 12 | not specified | inpatients daily isoniazid 300 mg, amithiozone 150 mg, and rifampicin 600 mg for at least 8 weeks; intramuscular pentamidine dimethansulphonate 4mg/kg for 15 alternate days | 12 patients 6 in the RH group and 6 pentamidine group  Daily RH group took isoniazid 300 mg, amithiozone 150 mg, and rifampicin 600 mg for at least 8 weeks as an inpatient  Pentamidine 4mg/kg QOD fifteen doses | One case in the RH group responds at eight weeks, and scar without inflammation at 12 weeks using biopsy, but five did not show parasitological or clinical improvement after eight weeks  RH was not effective for CL | Six cases showed marked clinical improvement at four weeks and the skin smear was negative after 15 doses of pentamidine |

Note: CL: Cutaneous leishmaniasis; LCL: Localized cutaneous leishmaniasis; DCL: Diffuse cutaneous leishmaniasis; IM: Intramuscular; IV: intravenous; SSG: Sodium stibogluconate; QOD: every other day; RH: Isoniazid and Rifampicin
