# Supplementary material for: Treatment outcomes of cutaneous leishmaniasis due to Leishmania aethiopica: A systematic review and meta-analysis
Source: PLoS One. 2023 Nov 2;18(11):e0293529. doi: 10.1371/journal.pone.0293529 (PMC10621858; doi:10.1371/journal.pone.0293529)
Supplement: S1 Text — (DOCX) [file pone.0293529.s004.docx]

**S1 Text**. Search strategy

1. PubMed

| Query | Filters | Search Details | Results |
| --- | --- | --- | --- |
| (("Leishmaniasis" OR "Oriental sore" OR "Cutaneous Leishmaniasis" OR "Diffuse Cutaneous Leishmaniasis" OR "Old World Cutaneous Leishmaniasis" OR "Mucocutaneous Leishmaniasis" OR "L. aethiopica" OR "Leishmania aethiopica") AND ("Cryotherapy" OR "Heat therapy" OR "Thermotherapy" OR "Systemic therapy" OR "Localized therapy" OR "Oral therapy" OR "Miltefosine" OR "Sodium stibogluconate" OR "Antimoniate" OR "Glucantime" OR "Meglumine antimoniate" OR "Liquid nitrogen therapy" OR "Paromomycin" OR "Amphotericin b" OR "Withholding treatment" OR "Conservative treatment" OR "Laser therapy")) AND ("Treatment outcome*" OR "Treatment failure" OR "Treatment Cure" OR "Relapse" OR "Unresponsiveness" OR "Non-responsiveness" OR "Failure" OR "Cure" OR "Outcome" OR "Partial response" OR "Dropout" OR "Treatment extension" OR "Resistance") | Abstract, Humans, English | (("Leishmaniasis"[All Fields] OR "Oriental sore"[All Fields] OR "Cutaneous Leishmaniasis"[All Fields] OR "Diffuse Cutaneous Leishmaniasis"[All Fields] OR "Old World Cutaneous Leishmaniasis"[All Fields] OR "Mucocutaneous Leishmaniasis"[All Fields] OR "l aethiopica"[All Fields] OR "Leishmania aethiopica"[All Fields]) AND ("Cryotherapy"[All Fields] OR "Heat therapy"[All Fields] OR "Thermotherapy"[All Fields] OR "Systemic therapy"[All Fields] OR "Localized therapy"[All Fields] OR "Oral therapy"[All Fields] OR "Miltefosine"[All Fields] OR "Sodium stibogluconate"[All Fields] OR "Antimoniate"[All Fields] OR "Glucantime"[All Fields] OR "Meglumine antimoniate"[All Fields] OR "Liquid nitrogen therapy"[All Fields] OR "Paromomycin"[All Fields] OR "Amphotericin b"[All Fields] OR "Withholding treatment"[All Fields] OR "Conservative treatment"[All Fields] OR "Laser therapy"[All Fields]) AND ("treatment outcome*"[All Fields] OR "Treatment failure"[All Fields] OR "Treatment Cure"[All Fields] OR "Relapse"[All Fields] OR "Unresponsiveness"[All Fields] OR "Non-responsiveness"[All Fields] OR "Failure"[All Fields] OR "Cure"[All Fields] OR "Outcome"[All Fields] OR "Partial response"[All Fields] OR "Dropout"[All Fields] OR "Treatment extension"[All Fields] OR "Resistance"[All Fields])) AND ((fha[Filter]) AND (humans[Filter]) AND (english[Filter])) | **1,261** |

1. Scopus

TITLE-ABS-KEY ((("leishmaniasis" OR "oriental sore" OR "cutaneous leishmaniasis" OR "diffuse cutaneous leishmaniasis" OR "old world cutaneous leishmaniasis" OR "mucocutaneous leishmaniasis" OR "l. aethiopica" OR "leishmania aethiopica" ) AND ( "cryotherapy" OR "heat therapy" OR "thermotherapy" OR "systemic therapy" OR "localized therapy" OR "oral therapy" OR "miltefosine" OR "sodium stibogluconate" OR "antimoniate" OR "glucantime" OR "meglumine antimoniate" OR "liquid nitrogen therapy" OR "paromomycin" OR "amphotericin b" OR "withholding treatment" OR "conservative treatment" OR "laser therapy" ) ) AND ( "treatment outcome*" OR "treatment failure" OR "treatment cure" OR "relapse" OR "unresponsiveness" OR "non-responsiveness" OR "failure" OR "cure" OR "outcome" OR "partial response" ) ) AND ( LIMIT-TO ( DOCTYPE , "ar" ) ) AND ( LIMIT-TO ( LANGUAGE , "english")) =**1932**

1. ScienceDirect

("Localized Cutaneous Leishmaniasis" OR "Mucocutaneous Leishmaniasis" OR "Diffuse Cutaneous Leishmaniasis" OR "Old World Cutaneous Leishmaniasis" OR “Leishmania aethiopica”) AND ("Treatment relapse" OR "Treatment failure" OR "Treatment Cure" OR “Partial response”)=**283**
