# Supplementary material for: Treatment outcomes of cutaneous leishmaniasis due to Leishmania aethiopica: A systematic review and meta-analysis
Source: PLoS One. 2023 Nov 2;18(11):e0293529. doi: 10.1371/journal.pone.0293529 (PMC10621858; doi:10.1371/journal.pone.0293529)
Supplement: Attachment — Submitted filename: Point by point response.pdf [file pone.0293529.s005.pdf]

Comments for Authors

The authors conducted a study on treatment outcomes of cutaneous leishmaniasis due to Leishmania aethiopica: A systematic review and meta-analysis. This is a very interesting and relevant study, but I suggest major changes.

Title

- similar study was done in 2016 (Treatment of Cutaneous Leishmaniasis Caused by Leishmania aethiopica: A Systematic Review by Johan van Griensven, Endalamaw Gadisa, Abraham Aseffa, Asrat Hailu, Abate Mulugeta Beshah and Ermias Diro). So what is new?
- Author affiliations: check the affiliation of Author one (Debre Tabor and Gondar Universities?)

Introduction

- Reference 1: Cutaneous leishmaniasis is a category one emergent uncontrolled neglected tropical skin disease (skin NTD) caused by obligate intracellular protozoa; Leishmania [1]. Correct as Cutaneous leishmaniasis (CL).-------------and correct Leishmania as Genus leishmania
- It would be better to write the introduction from General to specific
- You mentioned Predictors of outcome in the result section. So please include information about predictors on the introduction section

Materials and Methods

Eligibility criteria

We included studies on human being that reported treatment outcomes of CL due to L. aethiopica, published in English but without restriction to year of publication and study design. Already included in the above mentioned study, why do not you focus on the recent literatures and --------------

What about case reports, narrative review with no full information, qualitative studies and soon????

Inclusion and exclusion criteria---Very shallow

Data extraction and quality assessment: where is it?

The following data items were extracted: 1) primary author and year of publication; 2) study design; 3) diagnosis confirmation method; 4) sample size; 5) clinical phenotype of CL (LCL, MCL and DCL); 6) treatment detail (route, dose, dosage and treatment type); 7) characteristics of participants; and 8) treatment outcomes. Where is your data that contains the above variables????

Results

- Characteristics and quality of included studies. It better to replace by Description of included studies and Characteristics of the included studies subtitles and state accordingly
- Fig. 1 Flow chart of studies' search and retrieval process should be presented in the main document
- In Fig.1, report sought retrieval (n=53) and of them 33 studies excluded =when we sum up the number of studies excluded are 19, 19 vs 33????
- Five studies included from a reference list of articles excluded with reason. Why do you include those studies manually at the beginning in parallel with electronic searching engines
- Where is your Subgroup analysis result
- Clearly indicate trim and fill analysis for publication bias
- What models you used to assess heterogeneity
- Tables and figures should be presented in the result section of the main document
- Show your Subgroup and sensitivity analysis result in the main document

Discussion

- Compare your finding with another systemic studies and or guidelines. If not available you can compare with studies with large sample size
- Please discus variables used in subgroup analysis like year…
- Discus on Predictors of outcome

References

Write your references following standard Vancouver referencing style

In general: the title itself done previously, I found a lot of errors particularly in the methodology section, topographical errors and also I did not found new compared to the published.
