# Supplementary material for: Treatment outcomes of cutaneous leishmaniasis due to Leishmania aethiopica: A systematic review and meta-analysis
Source: PLoS One. 2023 Nov 2;18(11):e0293529. doi: 10.1371/journal.pone.0293529 (PMC10621858; doi:10.1371/journal.pone.0293529)
Supplement: Attachment — Submitted filename: Manuscript_V3.pdf [file pone.0293529.s009.pdf]

Treatment outcomes of cutaneous leishmaniasis due to *Leishmania aethiopica*: A systematic review and meta-analysis

Abebaw Yeshambel Alemu[1,2,3*], Lemma Derseh[2], Mirgissa Kaba[4], Endalamaw Gadisa[3], Kassahun Alemu[2]

Author affiliations

[1] Department of Pediatrics and Neonatal Health Nursing, College of Health Sciences, Debre Tabor University, Debre Tabor, Ethiopia

[2] Department of Epidemiology and Biostatistics, Institute of Public Health, College of Medicine and Health Sciences, University of Gondar, Gondar, Ethiopia

[3] Armauer Hansen Research Institute, Addis Ababa, Ethiopia

[4] School of Public Health, Addis Ababa University, Addis Ababa, Ethiopia

*__Corresponding author__: e-mail: yeshambelabebaw@gmail.com (AYA

Abstract

Background: *Leishmania aethiopica* is a unique species that causes cutaneous leishmaniasis, and studies evaluating the effectiveness of treatment for this condition reported inconsistent findings. This study was aimed at pooling evidence on treatment outcomes of CL due to *Leishmania aethiopica* to support decisions or propose further study.

Methods: We searched the main databases PubMed, Scopus and ScienceDirect, on the last date of 03 December 2022. A grey literature search in Google scholar and a manual search of the reference list of articles were performed. Next, screening, selection and critical appraisal of articles as well as data extraction were conducted. In addition to the narrative synthesis of evidence, and we performed meta-analysis using the *metaprop* package in Stata 17. The forest plots were constructed using a random effects model. The I-squared test and forest plots were

used to assess heterogeneity. We did sensitivity and subgroup analysis to address the heterogeneity. We assessed publication bias using a funnel plot and Egger's regression test.

Results: Twenty-two studies that involved 808 participants were reviewed, of which seven studies that comprised 677 participants were used in the meta-analysis. Though one study reported on combination therapy, the majority were antimonial monotherapy. The pooled proportion of cure was 63% (95% confidence interval (CI); 38-86%). In the subgroup analysis, the proportion was 61% (95% CI: 29-89 with systemic antimonial monotherapy, 87% (95% CI: 67-99%) with local therapy and 87% (95% CI: 78-93%) with combination therapy. While only localized cutaneous leishmaniasis (LCL) had shown a better cure with local therapy, all clinical phenotypes respond better with combination therapy. Across all clinical phenotypes, the pooled proportion of partial response was 19% (95% CI:14-25%), relapse 17% (95% CI: 12-22%), dropout 19% (95% CI:13-25%), and unresponsiveness was 6% (95% CI:3-9%).

Conclusions: The proportion of cure was low with antimonial monotherapy. Compared to antimonial monotherapy, combination therapy has a better clinical phenotype cure rate. Hence, combination therapy could be the potential choice of treatment across all clinical phenotypes of CL due to *L. aethiopica*, but effectiveness should be evaluated using high quality randomized control trials.

Keywords: Cure, Combination therapy, *Leishmania aethiopica*; Meta-analysis

Introduction

Cutaneous leishmaniasis (CL) is a category one emergent uncontrolled neglected tropical skin disease (skin NTD) caused by obligate intracellular protozoa; genus *Leishmania* [1]. Though more than 20 *Leishmania* species reported worldwide, *Leishmania tropica* (*L .tropica*), *Leishmania major* (*L .major*) cause most CL in Asia, Europe and Africa [2]. The unique species *Leishmania aethiopica* (*L. aethiopica*) is the cause of almost all CL in Ethiopia and Mount Elgon area of Kenya [3]. The main clinical phenotypes of CL due to *L. aethiopica* are localized cutaneous leishmaniasis (LCL), mucocutaneous leishmaniasis (MCL) and diffuse cutaneous

leishmaniasis (DCL) [4]. Amongst these clinical phenotypes, LCL is the commonest type, MCL is relatively common and DCL is a rare event [3].

The responses of CL to anti-leishmaniasis therapy vary by causative species[1, 4]. A meta-analysis of randomized control trial (RCT) studies showed high effectiveness photodynamic therapy forCL due to *L. major*, and thermotherapy works better than intramuscular pentavalent sodium stibogluconate (IM-SSG^V) for *L. tropica* [5]. Because of species level unresponsiveness, SSG^V is becoming ineffective to all *Leishmania* species [6].

According to the national leishmaniasis diagnosis and treatment guideline of Ethiopia, where almost all CL cases are due to *L. aethiopica*, withhold treatment, intralesional SSG^v and cryotherapy were recommended to treat LCL [7]. While systemic SSG^v was reported as ineffective in the guideline, systemic paromomycin was proposed as the potential treatment of choice for MCL and DCL. Additionally, a randomized phase II trial conducted in Amhara region Ethiopia showed 69% reduction in LCL lesion size at 16 weeks with four-weeks topical application of Shiunko ointment compared to only 22% in the placebo group [8]. Observational studies evaluating outcomes of CL due to *L. aethiopica* report cure rates ranging from 12.5% to 85% across different clinical phenotypes [9-12]. Unfavourable treatment outcomes, such as relapse, change in treatment regimen, treatment extension and failure also showed variation in Ethiopia [10-13]. Three Kenyan *L. aethiopica* confirmed cases treated with high dose (18 to 20mg/kg twice daily) of intravenous (IV) SSG^V for 30 days had shown disappearance of parasite from skin smear and culture after 14 to 27 days, with clinical healing of lesion and no recurrence at 3 to 18 months follow-up [14]. There are also studies which assessed treatment outcomes of CL due to *L. aethiopica* among immigrants. A Belgian traveller returned from northern Ethiopia had complete resolution of lesion after 45 days of intramuscular meglumine antimonate treatment [15].

Studies done in Ethiopia report predictors of treatment outcome for CL due to *L. aethiopica* [12, 16]. Among LCL cases treated with six cycles of intralesional (IL)-SSG^v, high chance of cure was positively associated with being male, increasing age and skin slit smear (SSS) grade +1 and +2 [16]. With 28 days of oral milteforsine treatment, MCL cases showed lower odds of relapse at

day 180 [12]. LCL cases treated with six cycles of IL-SSG [16] and all clinical phenotypes treated with 28 days oral miltefosine showed low chance of cure with increase lesion size [12].

Randomized control trial that assessed the effectiveness of different treatment options hardly exist to base treatment decision of CL due to *L. aethiopica []*. Besides, observational studies and very few non-randomized clinical trials that reported treatment outcomes of CL due to *L. aethiopica* revealed inconsistent findings to base clinical decisions. Therefore, the aim of this systematic review and meta-analysis was to summarize available evidence on the treatment outcomes of CL due to *L. aethiopica* to support clinical decision-making and suggest potential treatment alternatives for future randomized control trial.

Materials and Methods

Reporting

The protocol of this systematic review was registered in the PROSPERO database [**CRD42022306698]**. We used the Preferred Reporting Items for Systematic Review and Meta-analysis (PRISMA) checklist 2020 [17] to report the results (**S1 Checklist**).

Eligibility criteria

We included studies on human being that reported the responses of *L. aethiopica* to anti-leishmaniasis therapy, published in English but without restriction to year of publication and study design. Additionally, we did not limit studies by country because imported cases of CL due *L. aethiopica* were reported in non-endemic countries. Hence, all articles reporting treatment outcomes of CL due *L. aethiopica* everywhere were included in this study. However, systematic reviews, comments to the editor, conference proceedings and available without full length article were excluded.

Search strategy

We developed the Mnemonic PEO (Problem, Exposure, Outcome) to organize searching. A comprehensive searching of PubMed, Scopus and ScienceDirect databases was done using

Boolean operators 'AND' and 'OR' as appropriate. In PubMed and Scopus, we used the search terms: "Leishmaniasis" OR "Oriental sore" OR "Cutaneous Leishmaniasis" OR "Diffuse Cutaneous Leishmaniasis" OR "Old World Cutaneous Leishmaniasis" OR "Mucocutaneous Leishmaniasis" OR "L. aethiopica" OR "Leishmania aethiopica" AND "Cryotherapy" OR "Heat therapy" OR "Thermotherapy" OR "Systemic therapy" OR "Localized therapy" OR "Oral therapy" OR "Miltefosine" OR "Sodium stibogluconate" OR "Antimoniate" OR "Glucantime" OR "Meglumine antimoniate" OR "Liquid nitrogen therapy" OR "Paromomycin" OR "Amphotericin b" OR "Withholding treatment" OR "Conservative treatment" OR "Laser therapy" AND "Treatment outcome*" OR "Treatment failure" OR "Treatment Cure" OR "Relapse" OR "Unresponsiveness" OR "Non-responsiveness" OR "Failure" OR "Cure" OR "Outcome" OR "Partial response" OR "Dropout" OR "Treatment extension" OR "Resistance". Additionally, the following key terms were used to search in ScienceDirect: "Localized Cutaneous Leishmaniasis" OR "Mucocutaneous Leishmaniasis" OR "Diffuse Cutaneous Leishmaniasis" OR "Old World Cutaneous Leishmaniasis" OR "Leishmania aethiopica" AND "Treatment relapse" OR "Treatment failure" OR "Treatment Cure" OR "Partial response". The detailed searching strategy for the major databases is available in the **S1 Text**. To access grey literature, we searched the first 20 hits in Google scholar using the review title. Moreover, manual searching of reference lists was done.

Study selection process and quality assessment

All studies identified from databases and grey literature search were imported into Endnote X8 to remove duplicates. Two independent reviewers (AYA and KA) did the screening and selection of studies based on the title and abstract. These two reviewers did the critical appraisal of full-text articles. The Newcastle Ottawa Scale (NOS) [18] and risk of bias assessment tool version 2 (RoB2) [19] were used to appraise observational and clinical trial studies, respectively. The NOS results for observational studies was presented in Table A in **S1 Table,** and the RoB2 result was presented in Table B in **S1 Table**. All differences throughout the process were resolved through discussion. We adapted and used the PRISMA 2020 flow diagram to present the study selection process [17].

Data extraction and outcomes

We developed Microsoft excel sheet to extract the data. The excel was developed based on PRISMA template, and piloted before use. Two reviewers (AYA and KA) extracted the data.

The following data items were extracted: 1) primary author and year of publication; 2) study design; 3) diagnosis confirmation method; 4) sample size; 5) clinical phenotype of CL (LCL, MCL and DCL); 6) treatment detail (route, dose, dosage and treatment type); 7) characteristics of participants; and 8) Treatment outcomes. The results for these data items were available in Table A to C in **S2 Table**. We classified treatment outcomes as primary and secondary. While favourable outcomes were considered as primary, unfavourable outcomes were considered secondary.

Data analysis

We did narrative synthesis of evidence and meta-analysis. In addition to narrative synthesis of evidence, we did meta-analysis of proportions for treatment outcomes, in spite of the clinical and methodological heterogeneity between the included studies [20]. Meta-analysis was done using *metaprop* package in Stata 17. Since this package uses Freman-Tukey Transformation (ftt), the study specific and pooled confidence intervals (CI) were admissible and proportions close to 0% or 100% were included in the meta-analysis [21]. The pooled proportions of cure, partial response, relapse, dropout and unresponsiveness reported by two or more studies were estimated using random effects model and presented in forest plot [22].

To assess heterogeneity, I-squared statistics <75% and overlap confidence intervals on the forest plot were used [23]. We did subgroup analysis and sensitivity analysis to address high heterogeneity. While sensitivity analysis was presented graphically, we did subgroup analysis by study design, clinical phenotype of CL and type of treatment. Moreover, we assessed small study effect subjectively using funnel plot, and objectively using Egger's test.

Results

Search results

A total of 3520 studies were identified from all sources. We retrieved 52 studies pertinent to our objective. Of the 52 studies, 33 excluded, because of reasons mentioned in the PRISMA flow diagram (**Fig 1**). Then, 22 studies reporting treatment outcomes of CL due to *L. aethiopica* were included in the systematic review, of which seven studies were used in the meta-analysis.

Fig 1. Study selection using PRISMA flow diagram

Description and quality of included studies

We critically appraised 22 studies [8-12, 14-16, 24-37]. Twelve studies (nine case series [14, 24-26, 28-30, 32, 33] and three case reports [15, 34, 36]) documented treatment outcome as part of routine clinical practice. All case series and case reports scored less than five using NOS. Seven observational studies, five cohort studies [9, 10, 12, 16, 35] and two retrospective chart reviews [11, 37], scored five and above in the NOS. Table A in **S1 Table** showed the NOS of all observational studies included in the systematic review. Besides, three studies were clinical trials [8, 27, 31], but only one has low RoB [8] (Table B in **S1 Table**). Eighteen studies were done in Ethiopia [8-12, 16, 24-29, 31-33, 35-37], three studies were done among migrants and travelers [15, 30, 34], and one study was done in Kenya [14]. A total of 808 individuals participated in the 22 studies, of which 677 individuals participated in seven studies [9-12, 16, 35, 37] were used in the meta-analysis. Sixty-six individuals participated in three clinical trials [8, 27, 31], and 12 studies (nine case series [14, 24-26, 28-30, 32, 33] and three case reports [15, 34, 36]) involved 65 participants.

Parasitological confirmation of CL was done using skin slit smear (SSS) in 16 studies [8-12, 14, 16, 24, 25, 27, 30, 32-37]. The clinical phenotype of CL was reported in 17 studies [8, 9, 11, 12, 15, 16, 24, 26-29, 31-33, 35-37] but unspecified in five [10, 14, 25, 30, 34]. Accordingly, LCL was the commonest clinical phenotype documented in ten studies [8, 9, 11, 12, 15, 16, 31, 33, 35, 37]. Antimonial alone was the main treatment option documented across the studies, but eight studies [15, 25, 29, 30, 32, 35-37] report combination therapy. The details of study designs, sample size, confirmation of diagnosis, treatment type, dose and dosage, patient characteristics, responses to anti-leishmaniasis therapy were presented in **S2 Table**.

Treatment outcomes

In this study, primary outcomes such as cure, improved, negative/decrease in parasite density, good response, decrease in the size of lesion, and resolution of the lesion without scar were favourable outcomes. Asides, secondary outcomes such as recurrence, resistance, treatment

extension, partial response, relapse, dropout and unresponsiveness, progression to more severe stage and toxicity were unfavourable outcomes. Furthermore, this study also uncovered the predictors of treatment outcome.

Favourable outcomes

As shown in Table A in **S2 Table**, six Ethiopian cases with concomitant lesion (CL and leprosy) treated with systemic pentamidine responded well, but the authors did not report the route, dose and duration of treatment, and nothing documented how this outcome was ascertained [25]. Two prospective cohort studies also report positive treatment outcomes [9, 12] (Table B in **S2 Table**). IM-Meglumine antimonate (MA) resistant cases treated with 4m/kg IM pentamidine every-other-day (QoD) for 20 days had shown clinical and microscopy cure at six months [9]. After 100-150 mg oral miltefosine daily treatment, good clinical improvement reported in 70%, 28% and 14.3% of cases at day 28, day 90 and day 180 follow-up, respectively [12]. In a prospective follow-up study, 14% of LCL patients treated with six doses of IL-SSG^{V} showed good response at day 90 [16]. As shown in Table A in **S2 Table**, five Kenyans treated with high (18-20mg/kg twice daily) dose of IV-SSG^{V} were culture and smear negative at 14-27 days and 3-18 months follow-up [14]. Treatment outcomes were also reported among travelers and immigrant cases [15, 30, 34]. A Belgian traveller who returned from Ethiopia treated with 1.5 mg/5ml IM-MA for 45 days showed clinical resolution at 45 days and no relapse at six months [15]. An immigrant from Eretria to Germany treated with 200 mg/day IV-amphotericin B for 22 days healed at 12 months of follow-up without recurrence [34]. Three Ethiopian migrants in Israel had shown parasite clearance (smear and culture negative), clinical improvement and complete healing without scar at 10 days, 20 days, and 40 days after treatment, respectively, with 15% paromomycin sulfate and 12% methyl benzethonium chloride combination ointment [30].

Three clinical trials done in Ethiopia reported positive treatment outcomes (Table C in **S2 Table**). A phase II clinical trial compared the efficacy of four weeks twice daily Shiunko ointment with placebo among 20 LCL cases in each group. Five cases in the Shiunko group and four in the placebo group were cured. Six cases responded partially in both groups. Additionally, the Shiunko group showed 69% reduction in lesion size but only 22% in placebo group at 16 weeks

[8]. Among twelve cases (six in each group), a daily dose of three combination (isoniazid 300 mg, amithiozone 150 mg, and rifampicin 600 mg) drugs for eight weeks was compared with fifteen doses of 4mg/kg IM QoD pentamidine. In the combination group, one case improved without inflammation and scar at 12 weeks, and six cases in pentamidine group had negative smear at four weeks. In this trial, the clinical phenotype of CL was not reported [27]. Another clinical trial compared 200 mg itraconazole daily dose with placebo in 14 patients (four DCL and 10 LCL cases), seven in each group. No difference between the two group after four weeks [31].

Four case series studies [24, 28, 29, 32] documented positive treatment outcomes among DCL cases. Among 33 cases, cure was recorded in seven with pentamidine, three with chloroquine, one with primaquine and one with Amphotericin B (Table A in **S2 Table**). Yet, the dates of outcome measure were not specified [24]. In other study, two DCL cases treated with 4 mg/kg IM pentamidine daily for 2 weeks showed decrease in parasite number, but the date of outcome measure was not reported. The test of cure was done using smear microscopy and biopsy [28]. Similarly, three DCL cases treated with 1g 25% Chlorpromazine and 25% 50g Vaseline ointment for one month showed disappearance of clinical signs of inflammation in one case and decreased lesion size in another [29]. Two cases treated with 14 mg/kg IM-aminosidine daily for 60 days and one case treated with a combination of this same dose of Aminosidine plus 10 mg/kg IM-SSG daily. All cases got cure after two months of extended treatment [32].

Unfavourable outcomes

In Ethiopia, two CL-HIV co-infected cases treated with 20 mg/kg/day IV-SSG^V for 30 days had recurrence at five months of follow-up [33]. A case of MCL progressed to DCL after 30 days treatment with a combination of 20 mg/kg/day IM-SSG^V and 15 mg/kg/day paromomycin, and IM-SSG^V alone for extra 60 days [36]. No change to lesion observed in five MCL cases treated with four to eight weeks 500 mg oral metronidazole, and two cases report burning sensation [26]. A series of 33 DCL patients were unresponsive and had experienced toxicity for various treatment options (Table A in **S2 Table**). In this series, more than half of the cases were unresponsive to pentamidine (n=24) and pentostam (n=17). High toxicity (3+) was recorded in

pentamidine and amphotericin B treatment [24]. While five LCL cases got worse of their lesion, two cases were unresponsive to IL-SSG^{V} in a cohort study [16].

Clinical trial studies also reported negative outcomes (Table C in **S2 Table**). In a phase II clinical trial that compared Shiunko ointment versus placebo, six, eight and four of the twenty LCL cases showed partial response, treatment failure and dropout, respectively [8]. Similarly, after four weeks of treatment with 50 mg oral itraconazole versus placebo, five of the seven cases had active CL lesion at follow-up visit [31]. In a study that compared a combination of 300 mg isoniazid, 150 mg amithiozone and 600 mg rifampicin versus pentamidine five of the six cases showed parasitological or clinical improvement after eight weeks of daily treatment [27].

Predictors of outcome

This study showed that predictors of treatment outcomes were not investigated exhaustively. Only two cohort studies in Ethiopia report predictors of CL treatment outcome [12, 16]. Among LCL cases treated with IL-SSG^{V}, high probability of cure at day 90 was associated with older age, SSS grade +1 and +2 and being male [16]. In a cohort study that assessed outcome of patients treated with 150mg/day oral miltefosine for one month, MCL predicted low chance of relapse at day 180 compared to LCL [12]. Large lesion size decreases the chance of cure at day 90 in both studies [12, 16].

Meta-analysis

Seven studies [9-12, 16, 35, 37], all from Ethiopia, were included in the meta-analysis to estimate the pooled proportions of treatment outcomes of CL due to *L. aethiopica*. All studies included in this meta-analysis scored five and above with the NOS. Though the subjective judgment of funnel plot showed asymmetry (**Fig 2**), the insignificant statistical test with Egger's test (p-value= 0.329) indicated no publication. We estimated the pooled proportions of various treatment outcomes based on the meta-analysis of observational studies. However, high quality clinical trials that evaluate the effectiveness of available treatment options are lacking.

Fig 2. Funnel plot

Proportion of cure

The pooled proportion of cure estimated from seven studies [9-12, 16, 35, 37] was 0.63 (95% CI; 0.38-0.86; I^2=97.81%) (**Fig 3**). This pooled proportion did not consider cure rate after treatment extension, retreatment or change in treatment regimen. The studies included in the meta-analysis reported proportion of cure at different time point. The proportion ranged 55-95% at day 90 [12, 16, 35, 37], and 62-69% at day 180 [9, 12]. van Henten S. et al [12] reported 18.2% cure rate at day 28.

Fig 3. Forest plot of the pooled proportion of CL treatment cure

Subgroup and sensitivity analysis

Since the I-square statistic was high (97.81%), we did subgroup analysis by study design, treatment type and clinical phenotype for the pooled proportion of cure. By study design, the proportion was 0.74 (95% CI: 0.57-0.91; I^2=95.8%) in cohort studies, and 0.29 (95% CI: 0.22-0.35; I^2=. %) in retrospective chart reviews (**Fig 4**) but heterogeneity remain unresolved.

Fig 4. Subgroup analysis forest plot of treatment cure by study design

The proportion estimated from five studies [9-11, 35, 37] was 0.61 (95% CI: 0.29-0.89; I^2=96.68%) with systemic antimonial treatment (**Fig 5**). With local therapy, the proportion estimated from four studies [10, 16, 35, 37] was 0.87 (95% CI: 0.67-0.99; I^2=91.4%). Only one cohort study report cure with combination therapy as initial treatment, and the overall cure rate was 94.8% across all combination treatment options indicated [35]. All cases treated with local therapy alone had LCL clinical phenotype [10, 16, 35, 37]. The types of local therapy were IL-SSG^V [16, 37], cryotherapy [10] and combination of the IL-SSG^V and cryotherapy [35].

Fig 5. Subgroup analysis forest plot of treatment cure by treatment type

As shown in **Figure 6**, we did subgroup analysis across clinical phenotype (LCL, MCL and DCL). Accordingly, five studies [11, 12, 16, 35, 37] reported cure for LCL cases, and the estimated pooled proportion was 0.54 (95% CI: 0.18-0.88; I^2=96.73%). In two studies LCL cases were treated with IM-SSG^V treatment [11, 37], and with IL-SSG^V in the other [16]. Fikre et al

[11] documented unknown outcome in 35 LCL cases treated with IM-SSG^{V}. Additionally, the proportion of cure was 92.3% with cryotherapy alone, 96% with a combination of IL-SSG^{V} and cryotherapy, and 94% overall cure rate [35]. As such, four out of twelve (19%) LCL cases were cured with oral miltefosine [12].

Fig 6. Subgroup analysis forest plot of treatment cure by clinical phenotype

The proportion of cure for MCL cases estimated from four studies [11, 12, 35, 37] was 0.49 (95% CI: 0.21-0.77; I^2=91.68%) (**Fig 6**). Local treatment with cryotherapy was indicated in none of the studies [11, 12, 35, 37]. In two studies, [11, 37] the proportion ranged from 19-55% with IM-SSG^{V} treatment, but the outcome was unknown in 23 cases [11]. Furthermore, while 79% of the MCL cases treated with a combination of IM-SSG^{V} and Allopurinol, and 85.7% with IM-SSG^{V} and IL-SSG^{V} had shown cure, the overall MCL cure was 82% [35].

Moreover, the proportion of cure among DCL cases estimated from four studies was 0.51 (95% CI: 0.23-0.79; I^2=71.74%) (**Fig 6**). The cure rate of DCL cases with monotherapy (parenteral SSG^{V} or oral miltefosine) was below 50% [11, 12, 37]. In one of the studies [35], 80% DCL cases had shown cure with a combination of IM-SSG^{V} and Allopurinol, 86% with a combination of IM-SSG^{V}, Cryotherapy and IL-SSG, and the overall cure rate 83%. The cryotherapy and IL-SSG^{v} was used to treat satellite lesion.

Sensitivity analysis

In the sensitivity analysis no study affected the overall pooled proportion of cure, but a study by van Henten S. et al [12] showed some level of influence on the overall cure rate (**Fig 7**).

Fig 7. Sensitivity analysis

Proportions of unfavourable outcomes

In a cohort study, unfavourable outcome reported as negative outcome (unspecified) was 15% with four weeks 20mg/kg/day IL-MA every 3 days treatment [9]. This study reported 28% resistance with four weeks 20 mg/kg/day IV-MA treatment. In addition, 52% of relapsed cases retreated with the same does of IV-MA for four weeks were not cured [9]. van Henten S. et al

[12] reported 32.3% relapse at day 180 with four week 50 mg daily oral miltefosine treatment. Furthermore partial response was 20.4% [12] and 25% [11]. The detail on treatment outcomes at different time points is presented in Table B in **S2 Table**.

The pooled proportions of negative outcomes (partial response, relapse, dropout and unresponsiveness) reported in two or more studies were estimated (**Table 1**). All I-square tests showed low (<75%) heterogeneity in the pooled estimate of negative outcomes.

Table 1.The pooled proportion of unfavourable outcomes

Type of unfavourable outcome	Pooled proportion (95% CI)	I-squared statistics
Partial response [11, 12, 16, 37]	0.19 (95% CI: 0.14-0.25)	40.71%
Relapse [9, 12]	0.17 (95% CI: 0.12-0.22)	0
Dropout [10, 12]	0.19 (95% CI: 0.13-0.25)	0
Unresponsiveness [10, 11, 16]	0.06 (95% CI: 0.03-0.09)	41%

Discussion

In this systematic review and meta-analysis, we noted that systemic antimonial alone or in combination, local (topical) and other combination therapies were used to treat CL case due to *L. aethiopica*. Less than two-third of the cases cured with these treatment options. This finding also showed that unfavourable outcomes, such as partial response, relapse, dropout and unresponsiveness were significant. The studies we reviewed report treatment outcomes of CL due to *L. aethiopica* to a range of treatment options. For example, a case series reported treatment outcomes assessing more than ten therapeutic options [24]. This might indicate unavailability of standardized treatment guideline for CL due to *L. aethiopica* that contributed to selection of various treatment options relying on individual expertise and experiences of the clinicians. in Ethiopia where almost all CL cases are due to *L. aethiopica,* the national leishmaniasis diagnosis and treatment guideline underpinned the use of unstandardized and non-evaluated therapeutic options [7]. Thus, the evaluation of available therapeutic options and standardize the treatment of CL due to *L. aethiopica* should be a national priority.

In this study, the overall pooled proportion of cure was 63% with available therapeutic options. This proportion was low with systemic antimonial monotherapy such as IV/IM-SSG[V] and high with local and combination therapies. However, paromomycin was proposed as the treatment of choice to treat CL due *L. aethiopica* in the national leishmaniasis diagnosis and treatment guideline of Ethiopia [7]. In our meta-analysis, high proportion of cure with local therapy was limited to LCL, but combination therapy works across all clinical phenotypes. The proportion was as low as 29% with systemic antimonies alone. Low cure rate might be due to low efficacy of antimonial drugs for CL due to *L. aethiopica*, and high drug resistance of this species. As such, the proportion of cure was only 61% with systemic antimonial monotherapy. In our review, resistance to systemic meglumine antimonate was 28% [9]. A systematic review also report about increasing resistance of *Leishmania* species for SSG[V] drug [4]. The Ethiopian leishmaniasis diagnosis and treatment guideline also report ineffectiveness of SSG to treat MCL and DCL due to *L. aethiopica*, but no evaluation done yet [7]. In our finding, despite low cure rate, SSG[V] was reported as the mainstay of treatment for CL due to *L. aethiopica*. This implies the necessity of future study that assess the resistance pattern of this protozoa for existing treatment options.

In the subgroup analysis, CL due to *L. aethiopica* had shown 87% cure rate with local therapy. This finding was in line with a systematic review [38] that report greater than 80% cure rate for CL due *L. major* and *L. tropica* with topical agents. Thermotherapy, paromomycin and combinations, CO_2 laser, 5-aminolevulinic acid hydrochloride (10%) plus visible red light (633 nm) and cryotherapy were used [38]. However, only liquid nitrogen-based cryotherapy and IL-SSG were used in our review [10, 16, 35, 37]. In all the studies were reviewed, IL-SSG was the main topical agent. The effectiveness of this topical agent alone was low. For example, in a cohort study we reviewed, the cure rate was 60% with IL-SSG [16]. Liquid nitrogen-based cryotherapy is expensive with an estimated cost of 4 USD per patient [10]. So, other topical agents such as thermotherapy, CO_2 laser and thermotherapy could be considered for CL due to *L. aethiopica*, but cost-effectiveness of these therapies require further study.

In this study, we found no significant difference in cure rate across the different clinical phenotypes. A cohort study also showed no significant difference in cure rate between DCL and

LCL cases [12]. However, in our review, combination of SSG^V with other treatment options showed high cure rate ranging from 78.6-96% across various clinical phenotypes [35].Accordingly, with a combination of IL-SSG^V and cryotherapy, the proportion of cure was 96% in LCL cases. In MCL cases, the proportion was 79% with a combination of IM-SSG^V and IL-SSG^V, and 85.7% with a combination of IM-SSG^V and Allopurinol. In DCL cases, the proportion of cure was 80% with a combination of IM-SSG^V and Allopurinol, and 86% with a combination of IM-SSG^V, cryotherapy and IL-SSG^V [35]. A systematic review also report 70-100% cure rate with a combination of pentavalent antimonial with other alternatives [39], but the species of leishmania and the clinical phenotypes were not reported. Thus, clinicians could consider combination therapies to increase cure rate of CL due to *L. aethiopica*, and combination therapy should be the option included in the national leishmaniasis diagnosis and treatment guideline.

The pooled proportion of unfavorable outcomes of CL due to *L. aethiopica* were partial response (19%), relapse (17%), dropout (19%) and unresponsiveness (6%). The proportion of relapse in our finding was higher than 2-6% relapse reported in systematic review that assessed effectiveness of different topical agents for LCL cases due to *L. major* and *L. tropica* [38]. High proportion of relapse in our finding might be due to severe clinical phenotypes such as MCL and DCL associated with *L. aethiopica* [3]. These severe clinical phenotypes are difficult to treat [40]. Similarly, partial response and unresponsiveness were unacceptably high in our finding. This might be due to high resistance of *L. aethiopica* for available treatment options; as described earlier. The dropout might also increase the probability of partial response, relapse and unresponsiveness. Hence, due account of drug resistance and reducing dropout should not be understated to counter negative outcomes.

This systematic review showed that being male, older age and SSS grade +1 and +2 were positively associated with high probability of cure among LCL cases treated with IL-SSG^v [16]. Increasing lesion size decrease the probability of cure across all clinical phenotypes treated with oral miltefosine [12] and LCL cases treated with IL-SSG^v [16]. In miltefosine treated cases, MCL cases had lower odds of relapse compared to LCL. However, these two studies had clinical

and methodological heterogeneity to pool the effect size. This calls for primary studies that identify predictors of treatment outcome in CL due *L. aethiopica*.

This systematic review and meta-analysis provided an updated evidence on treatment outcomes of CL due to *L. aethiopica* with available anti-leishmaniasis therapy. However, the study was not without limitations. The first limitation was heterogeneity persisted in the random effects model and subgroup analysis, but the summary estimates can be used for decision making with cautious interpretation, and evidence from two studies is more powerful for decision than evidence from a single clinical trial [41]. Second, the number of studies in the meta-analysis were below ten which might negatively affect the reliability of the funnel plot and Egger's test. Furthermore, clinical trial studies included in this study had low quality, involve small sample size, and none of the studies assessed the effectiveness of either local, combination or systemic antimonial therapy using high quality randomized control trial.

Conclusions

The pooled proportion of cure for CL due to *L. aethiopica* was low across all clinical phenotypes. Compared to antimonial monotherapy, local and combination therapies have shown better cure rate. While LCL, MCL, and DCL clinical phenotypes had higher cure rates with combination therapy, only LCL had a higher cure rate with local therapy. Additionally, the magnitude of unfavourable treatment outcomes such as partial response, relapse, dropout and unresponsiveness were significant. The effectiveness of neither local therapy nor combination therapy was evaluated for *L. aethiopca* treatment using high quality randomized clinical trial. Therefore, combination therapies across all clinical phenotypes and local therapy in LCL are potential choices to increase the rate of favourable outcomes. The effectiveness of combination therapy across all clinical phenotypes and local therapy in LCL should be evaluated using high quality randomized control trials.

Abbreviations: CL, Cutaneous Leishmaniasis; DCL, Diffuse Cutaneous Leishmaniasis; IM, Intramuscular; IV, Intravenous; LCL, Localized Cutaneous Leishmaniasis; MCL, Mucocutaneous Leishmaniasis; MA, Meglumine Antimonate; NOS, Newcastle Ottawa Scale; RoB, Risk of Bias Assessment; SSG^{V}, Sodium Stibogluconate; QoD, Every Other Day

Author contributions: AYA: Conceptualization, data curation, formal analysis, methodology, software, writing-original draft and writing-review and editing; **LD**: Data curation, methodology, writing-review and editing; **MK**: Data curation, methodology, writing-review and editing; **EG**: Data curation, methodology, writing-review and editing; **KA**: Data curation, methodology, writing-review and editing

Financial disclosure: The author(s) received no specific funding for this work. The Skin Health Africa Research Program project financially supported the first author in the form of student stipend, but the project did not decide in the design, analysis and reporting of this study.

Competing interests: The authors have declared that no competing interests exist.

Data Availability Statement: All relevant data are within the manuscript and its Supporting Information files.

Acknowledgement: Not applicable

References

1.de Vries HJC, Schallig HD. Cutaneous Leishmaniasis: A 2022 Updated Narrative Review into Diagnosis and Management Developments. Am J Clin Dermatol. 2022;23(6):823-40.

2.Akilov OE, Khachemoune A, Hasan T. Clinical manifestations and classification of Old World cutaneous leishmaniasis. International journal of dermatology. 2007;46(2):132-42.

3.van Henten S, Adriaensen W, Fikre H, Akuffo H, Diro E, Hailu A, et al. Cutaneous leishmaniasis due to Leishmania aethiopica. EClinicalMedicine. 2018;6:69-81.

4.McGwire BS, Satoskar AR. Leishmaniasis: clinical syndromes and treatment. QJM: An International Journal of Medicine. 2013;107(1):7-14.

5.Gonzalez U, Pinart M, Reveiz L, Alvar J. Interventions for Old World cutaneous leishmaniasis. Cochrane database of systematic reviews. 2008(4).

6.Madusanka RK, Silva H, Karunaweera ND. Treatment of cutaneous leishmaniasis and insights into species-specific responses: a narrative review. Infectious Diseases and Therapy. 2022:1-17.

7.Ministry of Health Ethiopia. Guideline for Diagnosis, Treatment and Prevention of Leishmaniasis in Ethiopia. In: Directorate AHPaDP, editor. 2nd Edition ed. Addis Ababa: FMoH; 2013.

8.Na-Bangchang K, Ahmed O, Hussein J, Hirayama K, Kongjam P, Aseffa A, et al. Exploratory, phase II controlled trial of Shiunko ointment local application twice a day for 4 weeks in Ethiopian patients with localized cutaneous leishmaniasis. Evidence-Based Complementary and Alternative Medicine. 2016;2016.

9.Padovese V, Terranova M, Toma L, Barnabas GA, Morrone A. Cutaneous and mucocutaneous leishmaniasis in Tigray, northern Ethiopia: clinical aspects and therapeutic concerns. Transactions of the Royal Society of Tropical Medicine and Hygiene. 2009;103(7):707-11.

10.Negera E, Gadisa E, Hussein J, Engers H, Kuru T, Gedamu L, et al. Treatment response of cutaneous leishmaniasis due to Leishmania aethiopica to cryotherapy and generic sodium stibogluconate from patients in Silti, Ethiopia. Transactions of the Royal Society of Tropical Medicine and Hygiene. 2012;106(8):496-503.

11.Fikre H, Mohammed R, Atinafu S, van Griensven J, Diro E. Clinical features and treatment response of cutaneous leishmaniasis in North-West Ethiopia. Tropical Medicine & International Health. 2017;22(10):1293-301.

12.van Henten S, Tesfaye AB, Abdela SG, Tilahun F, Fikre H, Buyze J, et al. Miltefosine for the treatment of cutaneous leishmaniasis—A pilot study from Ethiopia. PLoS neglected tropical diseases. 2021;15(5):e0009460.

13.Padovese V, Terranova M, Toma L, Barnabas GA, Morrone A. Cutaneous and mucocutaneous leishmaniasis in Tigray, northern Ethiopia: clinical aspects and therapeutic concerns. Transactions of the Royal Society of Tropical Medicine and Hygiene. 2009;103(7):707-11.

14.Chulay J, Anzeze E, Koech D, Bryceson A. High-dose sodium stibogluconate treatment of cutaneous leishmaniasis in Kenya. Transactions of the Royal Society of Tropical Medicine and Hygiene. 1983;77(5):717-21.

15.Mengeot L, Yombi J-C, Baeck M. Cutaneous leishmaniasis due to Leishmania aethiopica: A therapeutic challenge. JAAD Case Reports. 2022;20:72-5.

16.Tilahun Zewdu F, Tessema AM, Zerga AA, van Henten S, Lambert SM. Effectiveness of intralesional sodium stibogluconate for the treatment of localized cutaneous leishmaniasis at Boru Meda general hospital, Amhara, Ethiopia: Pragmatic trial. PLoS neglected tropical diseases. 2022;16(9):e0010578.

17.Page MJ, McKenzie JE, Bossuyt PM, Boutron I, Hoffmann TC, Mulrow CD, et al. The PRISMA 2020 statement: an updated guideline for reporting systematic reviews. BMJ. 2021;372:n71.

18.Peterson J, Welch V, Losos M, Tugwell P. The Newcastle-Ottawa scale (NOS) for assessing the quality of nonrandomised studies in meta-analyses. Ottawa: Ottawa Hospital Research Institute. 2011;2(1):1-12.

19.Cochrane Group. Cochrane Handbook for Systematic Reviews of Interventions. Current version of RoB 2. 2019 [Available from: https://www.riskofbias.info/welcome/rob-2-0-tool/current-version-of-rob-2.

20.Schulzke S. Assessing and Exploring Heterogeneity. Principles and Practice of Systematic Reviews and Meta-Analysis: Springer; 2021. p. 33-41.

21.Nyaga VN, Arbyn M, Aerts M. Metaprop: a Stata command to perform meta-analysis of binomial data. Archives of Public Health. 2014;72(1):1-10.

22.Deeks JJ, Higgins JP, Altman DG, Group CSM. Analysing data and undertaking meta-analyses. Cochrane handbook for systematic reviews of interventions. 2019:241-84.

23.Deshmukh M. Sensitivity and Subgroup Analyses. Principles and Practice of Systematic Reviews and Meta-Analysis: Springer; 2021. p. 89-97.

24.Bryceson A. Diffuse cutaneous leishmaniasis in Ethiopia: II Treatment. Transactions of the Royal Society of Tropical Medicine and Hygiene. 1970;64(3):369-79.

25.Barnetson RS, Bryceson A. Cutaneous leishmaniasis and leprosy. Transactions of the Royal Society of Tropical Medicine and Hygiene. 1978;72(2):160-3.

26.Belehu A, Naafs B, Touw-Langendijk E. Failure of metronidazole treatment in Ethiopian mucocutaneous leishmaniasis. Brit J Derm. 1978;99(4):421-2.

27.Van der Meulen J, Mock B, Fekete E, Sarojini P. Limited therapeutic action of rifampicin/isoniazid against Leishmania aethiopica. Limited therapeutic action of rifampicin/isoniazid against Leishmania aethiopica. 1981;2:197-8.

28.Zaar K, Wunderlich F, Belehu A. Electron microscopical studies on cutaneous leishmaniasis in Ethiopia: I. The diffuse form and its treatment with pentamidine. Annals of Tropical Medicine & Parasitology. 1982;76(6):595-605.

29.Henriksen T-H, Lende S. Treatment of diffuse cutaneous leishmaniasis with chlorpromazine ointment. The Lancet. 1983;321(8316):126.

30.Weinrauch L, Katz M, El On J. Leishmania aethiopica: topical treatment with paromomycin and methylbenzethonium chloride ointment. Journal of the American Academy of Dermatology. 1987;16(6):1268-70.

31.Akuffo H, Dietz M, Teklemariam S, Tadesse T, Amare G, Berhan TY. The use of itraconazole in the treatment of leishmaniasis caused by Leishmania aethiopica. Transactions of the Royal Society of Tropical Medicine and Hygiene. 1990;84(4):532-4.

32.Teklemariam S, Hiwot AG, Frommel D, Miko TL, Ganlov G, Bryceson A. Aminosidine and its combination with sodium stibogluconate in the treatment of diffuse cutaneous leishmaniasis caused by Leishmania aethiopica. Transactions of the Royal Society of Tropical Medicine and Hygiene. 1994;88(3):334-9.

33.Berhe N, Hailu A, Gemetchu T. Human immunodeficiency virus and recurrence of cutaneous leishmaniasis long after healed localized cutaneous leishmaniasis due to Leishmania aethiopica. Transactions of the Royal Society of Tropical Medicine and Hygiene. 1995;89(4):400-1.

34.Zanger P, Kötter I, Raible A, Gelanew T, Schönian G, Kremsner PG. Case report: Successful treatment of cutaneous leishmaniasis caused by Leishmania aethiopica with liposomal amphothericin B in an immunocompromised traveler returning from Eritrea. The American journal of tropical medicine and hygiene. 2011;84(5):692.

35.Seife T, Benecha A, Zewdu F, Ayal A, Misganaw M. Treatment patterns and effectivness of anti-leishmaniasis agents for patients with cutaneous leishmaniasis at Boru Meda Hospital, South Wollo, North East Ethiopia, 2017/18. J Clin Exp Dermatol Res. 2018;9(03).

36.Tegegne B, Alemu G. Progress of mucocutaneous leishmaniasis to drug nonresponsive diffuse cutaneous leishmaniasis in Ethiopia. A case report. International medical case reports journal. 2020;13:551.

37.Tesfa D, Manaye N, De Vries HJ, Van Griensven J, Enbiale W. Clinical pattern and treatment outcome of Cutaneous Leishmaniasis in two hospitals in Bahir Dar, Ethiopia (2017-2021). The Journal of Infection in Developing Countries. 2022;16(08.1):26S-34S.

38.Nassif PW, DE MELLO TFP, Navasconi TR, Mota CA, Demarchi IG, Aristides SMA, et al. Safety and efficacy of current alternatives in the topical treatment of cutaneous leishmaniasis: a systematic review. Parasitology. 2017;144(8):995-1004.

39.Berbert TRN, Mello TFPd, Wolf Nassif P, Mota CA, Silveira AV, Duarte GC, et al. Pentavalent antimonials combined with other therapeutic alternatives for the treatment of cutaneous and mucocutaneous leishmaniasis: A systematic review. Dermatology research and practice. 2018;2018.

40.Reithinger R, Dujardin J-C, Louzir H, Pirmez C, Alexander B, Brooker S. Cutaneous leishmaniasis. The Lancet Infectious Diseases. 2007;7(9):581-96.

41.Haidich A-B. Meta-analysis in medical research. Hippokratia. 2010;14(Suppl 1):29.

Supporting information

S1 Table. Quality of studies included in the systematic review and meta-analysis

S2 Table. Characteristics of studies, treatment details, participant characteristics and treatment outcomes

S1 Checklist. PRISMA check list

S1 Text. Searching strategy
