# Supplementary material for: Treatment outcomes of cutaneous leishmaniasis due to Leishmania aethiopica: A systematic review and meta-analysis
Source: PLoS One. 2023 Nov 2;18(11):e0293529. doi: 10.1371/journal.pone.0293529 (PMC10621858; doi:10.1371/journal.pone.0293529)
Supplement: Attachment — Submitted filename: Response to Reviewers.docx [file pone.0293529.s007.docx]

**Point by a point response letter**

Dear Academic Editor (Dr. Balew Arega Negatie, MD, MSc), Reviewer #1, and Reviewer #2

After going through the entire manuscript, you forwarded your constructive comments which we missed to touch. Therefore, we are glad enough to express our sincerest thanks for your constructive editorial and review comments that could help to improve the novelty of our effort. We first presented the comments and author response to the editor comments followed by Reviewer #1 and Reviewer #2, respectively. The comments were highlighted yellow and the author response highlighted pink.

**Point by point response to academic editor’s comment**

**Editor’s general comment**

Comment 1. Please ensure that your manuscript meets PLOS ONE's style requirements, including those for file naming. The PLOS ONE style templates can be found at

<https://journals.plos.org/plosone/s/file?id=wjVg/PLOSOne_formatting_sample_main_body.pdf>

<https://journals.plos.org/plosone/s/file?id=ba62/PLOSOne_formatting_sample_title_authors_affiliations.pdf>

Response: Thank you dear reviewer for your honorable comment. We followed the PLOS ONE author’s guideline to prepare the manuscript. The figures were prepared using PACE and uploaded separately. Additionally, we prepared the tables and supporting files as per the authors guideline.

**Additional Editor comments taken from tacked manuscript**

Comment: This is good if rephase like this “Leishmania aethiopica is a unique species that causes cutaneous leishmaniasis, and studies evaluating the effectiveness of treatment for this condition reported inconsistent findings

Response: Thank you dear editor for the suggestion. It has been revised as per the comment and the changes made are available in track change in the abstract section.

Comment: Change to “decisions “

Response: Thank you so much. It has been edited as per the comment in the revised document.

Comment: The narrative

Response: Thank you editor. We revised the grammar as per the comment.

Comment: Remove this sentences

Response: Thank you dear editor once again. The statement has been removed from the abstract section as per the comment and the change can be seen in the track change.

Comment: The conclusion is based on the single study finding which is not appropriate

Response: Dear editor you for the comment. We made revision as per the comment and the changes can be seen in track change in the conclusion section of the abstract.

Comment: This can be rephrased as “Compared to antimonial monotherapy, combination therapy has a better clinical phenotype cure rate”

Response: Thank you for the suggestion. We revised the conclusion as per the suggestion and comment. The revisions made can be seen in the conclusion section of the abstract.

Comment: Was reported

Response: Thank you once again. We revised as per the comment and the changes can be seen in track changes in the revised manuscript.

Comment: This paragraph is not related to the different species of Leishmania. So, either remove it totally or make it short.

Response: Thank you for your comment. We make the paragraph short than removing it. We did not remove it because the responses to anti-leishmaniasis therapy were our focus. So the existing knowledge about the difference in species level response to treatment was summarized. The response of L.tropica and L.major to different treatment was summarized.

Comment: Here, please writes the guideline-recommended treatment regimen in the country if any.

Response: Thank you dear editor for the comment.

Comment: “Summarizes” is a better word than the aggregate

Response: Thank you dear editor for your suggestion. We revised it accordingly and the changes can be seen in the introduction section at page

Comment: Why do you ignore the heterogenicity? How do you do MA while there is significant heterogenicity?

Response: Thank you dear editor. Yes, there were methodological and clinical heterogeneity between the studies that we reviewed, but this did not limit authors to perform meta-analysis [1]. Hence, we did met-analysis and checked statistical heterogeneity. Because we detected high I-square >75% we did subgroup analysis and sensitivity analysis and report these results with the main analysis for the readers. It is possible to conduct meta-analysis despite the methodological and clinical heterogeneity, and when statistical heterogeneity exists, we can do subgroup analysis based on the clinical and methodological variation.

Comment: See above comment, you already plan to ignore heterogenicity, what is the importance of this sentence and subsequent below?

Response:

Comment: “Favorable vs unfavorable” is better wording than positive vs negative

Response: Thank you dear editor. We revised as per the comment and the changes made can be seen in the track change in the results section.

Comment: In the presence of such high heterogenicity, what is the importance of determining the pooled estimate? It good simply narrate the finding in such a cases.

Response: Thank you dear editor. Yes of course combining apples and oranges is not advisable. Our interest was not to compare the effect of different treatment options rather we estimated the pooled estimates of response of *L. aethiopica* for anti-leishmaniasis therapy. When the researcher’s interest is to estimate the response of a disease for various treatment options, in general, it is possible to pool the effect size [2]. Furthermore, meta-analysis should be conducted when a group of studies is sufficiently homogeneous in terms of subjects involved, interventions, and outcomes to provide a meaningful summary. However, it is often appropriate to take a broader perspective in a meta-analysis than in **a single clinical trial** [3].

Comment: In Figure 5, the is only one study in the subgroup analysis (Seife T/2018). However, there must be at least two studies in each subgroup. The finding of this analysis skewed your conclusion and recommendation. This must be corrected and revise your conclusion accordingly.

Response: Thank you dear editor for your high-level curiosity and valid comment. Yes, there is only one study for the sub-group analysis by treatment type. The sub-group analysis was made using weighted random effects model. So, the result will not be skewed by Seife T et al /2018 because it accounts only for 10.27% in the sample weights. Since sub-group analysis is not primary analysis, this single study was isolated and weighted alone while the studies that report similar treatment type were grouped together. Besides, the pooled effect size in the sub-group analysis was not reported so that it would not skew the pooled estimate. Moreover, the narrative review report was presented together with the meta-analysis, so readers and decision makers can evaluate the finding.

Comment: These sentences should be modified. In the presence of high heterogeneity, what is the importance of determining the pooled estimate?

Response: Thank you dear editor. Yes of course there is no recommendation to conduct meta-analysis based on a single study, but in the subgroup analysis only one study can be remained as single when those studies that showed similar grouping variable are grouped together. The study by Seife T et al /2018 remained as single when the subgroup of studies that report cure with antimonial therapy and local therapy were grouped together. This single study result can be seen in the forest plot, but weighted for the sample size. The study by Seife T et al /2018 had 10.27% contribution.

Comment: Please support with a reference

Response: Thank you dear editor. We cited the reference as per your comment.

Comment: Is there any recommendation to pool effect from a single study? see other comments too.

Response: Thank you dear editor. Your concern is highly respected. Yes of course there is no recommendation to conduct meta-analysis based on single study. The pooled effect of a single study shown in the forest plot of sub-group analysis by treatment was not primary meta-analysis result that aimed to pool the cure rate from a single study.

Comment: See the comments about figure 5.

Response: Thank you indeed! The comment is highly valued. The case of Figure 5 was to show the contribution of combination therapy by Seife T et al /2018 in the sub-group analysis. While antimonial and local therapy incorporated more than one study, combination therapy remained with only one study. The conclusion was narrated based on the result from the review, and we did not include cure rate with percentage to avoid misleading the readers and decision makers.

**Point by point response to comments by Reviewer # 1**

**General comments to the Author by reviewer #1**

Comment 1. Is the manuscript technically sound, and do the data support the conclusions?

The manuscript must describe a technically sound piece of scientific research with data that supports the conclusions. Experiments must have been conducted rigorously, with appropriate controls, replication, and sample sizes. The conclusions must be drawn appropriately based on the data presented.

Reviewer #1: No

Response: Thank you dear reviewer. We revised the methodology for technical soundness of the study as per your comments. The revisions made can be depicted in track changes in the materials and methods section of the revised manuscript.

Comment 2. Has the statistical analysis been performed appropriately and rigorously?

Reviewer #1: No

Response: Thank you dear reviewer. We are grateful for your scholarly comments. The manuscript has been revised intensively as per your comments. The changes made can be seen in track changes throughout the manuscript.

Comment 3. Have the authors made all data underlying the findings in their manuscript fully available?

The PLOS Data policy requires authors to make all data underlying the findings described in their manuscript fully available without restriction, with rare exception (please refer to the Data Availability Statement in the manuscript PDF file). The data should be provided as part of the manuscript or its supporting information, or deposited to a public repository. For example, in addition to summary statistics, the data points behind means, medians and variance measures should be available. If there are restrictions on publicly sharing data—e.g. participant privacy or use of data from a third party—those must be specified.

Reviewer #1: Yes

Response: Thank you dear reviewer for your encouraging comment.

Comment 4. Is the manuscript presented in an intelligible fashion and written in standard English?

PLOS ONE does not copyedit accepted manuscripts, so the language in submitted articles must be clear, correct, and unambiguous. Any typographical or grammatical errors should be corrected at revision, so please note any specific errors here.

Reviewer #1: Yes

Response: Thank you dear reviewer for your encouraging comment.

**Specific review comments to the author by reviewer #1 taken from the attachment file**

Comment: The authors conducted a study on treatment outcomes of cutaneous leishmaniasis due to Leishmania aethiopica: A systematic review and meta-analysis. This is a very interesting and relevant study, but I suggest major changes.

Response: Dear reviewer thank you for your encouraging comment and indicating points for improvement of our work. Major revisions have been made as per your comments and suggestions as highlighted with track changes in the manuscript.

**Title**

Comment: similar study was done in 2016 (Treatment of Cutaneous Leishmaniasis Caused by Leishmania aethiopica: A Systematic Review by Johan van Griensven, Endalamaw Gadisa, Abraham Aseffa, Asrat Hailu, Abate Mulugeta Beshah and Ermias Diro). So, what is new?

Response: Thank you so much dear reviewer for your meticulous reading and comment. We accept your comment with great honor. However, our systematic review and meta-analysis generated an additional evidence to the existing review, and we generated the following new evidences to existing knowledge on the responses of L. aethiopica to anti-leishmaniasis therapy.

1. We estimated the pooled proportion of cure based on the meta-analysis of proportions of cure reported from seven studies, this might be taken as additional evidence. However, the previous article did not report the pooled proportion of cure.
2. The secondary outcomes of our systematic review and meta-analysis also revealed the pooled proportions of unfavorable outcomes, such as treatment failure, partial response, dropout and unresponsiveness. Nonetheless, the previous study did not pool evidences on these outcomes. We felt that these reports are also additional evidence to the scientific community to anticipate the proportions of unfavorable treatment outcomes while treating CL due to L. aethiopica.
3. Unlike the previous narrative review, the pooled proportion of cure was reported based on the clinical phenotype of CL and type of treatment. We felt that this is also additional evidence to the existing body of knowledge.
4. After systematic review done in 2016 eight studies published. We included these 8 new studies in our systematic review and meta-analysis that gave us the chance to pool the favorable and unfavorable responses of L. aethiopica to anti-leishmaniasis therapy, but his reports were impossible in the previous review because the studies were not enough to generate pooled evidence.
5. It has been seven years since the previous systematic review has been published, so our study generated more recent evidence. Additionally, I consulted a senior Biostatician and an Associate professor at University of Gondar about the possibility of doing systematic review and meta-analysis in the presence of published systematic review. He affirmed that new systematic review and meta-analysis required after five years to generate additional knowledge, provide new studies are added.

Comment: Author affiliations: check the affiliation of Author one (Debre Tabor and Gondar Universities?)

Response: Thank you for your curiosity comment. Yes of course it is not possible to get employment at two public institutions according to the Ethiopian government civil service rule and regulation. However, first author is a PhD student affiliated to University of Gondar, sponsored by Armauer Hansen Research Institute and the employee of Debre Tabor University. According the PhD guideline used by University of Gondar, publication by a PhD student should have affiliation to University of Gondar so that the University’s contribution is recognized. The same works for Armauer Hansen Research Institute too.

**Introduction**

Comment: Reference 1: Cutaneous leishmaniasis is a category one emergent uncontrolled neglected tropical skin disease (skin NTD) caused by obligate intracellular protozoa; Leishmania [1]. Correct as Cutaneous leishmaniasis (CL)-------------and correct Leishmania as Genus leishmania

Response: Heartful thanks for the suggestion. The revision has been made as can be seen in track changes in the revised document.

Comment: It would be better to write the introduction from General to specific

Response: Thank you dear reviewer. We revised the manuscript as per the comment and the changes can be seen in the track changes in the revised manuscript.

Comment: You mentioned Predictors of outcome in the result section. So please include information about predictors on the introduction section

Response: Sure! We included the predictors of treatment outcome in the introduction section. The additions and changes made can be seen in track change in the fourth paragraph of the introduction section.

**Materials and Methods**

**Eligibility criteria**

Comment: We included studies on human being that reported treatment outcomes of CL due to L. aethiopica, published in English but without restriction to year of publication and study design. Already included in the above-mentioned study, why do not you focus on the recent literatures and --------------

Response: Thank you dear reviewer. Yes, some of the studies were included in the previous systematic review, but those articles should not be removed because included in the past review. Besides one of the goals of conducting manual searching of the reference list of systematic reviews excluded is to include those studies included in that prior systematic review.

Comment: What about case reports, narrative review with no full information, qualitative studies and soon????

Response: Thank you once again. We revised as per the comment, and we included the revision in the eligibility criteria sub-section of the methods and materials section. The changes made can be seen in track changes.

Comment: Inclusion and exclusion criteria---Very shallow

Response: Thank you dear reviewer. We included the exclusion and inclusion criteria as per the comments and the changes made can be seen in the eligibility criteria sub-section.

Comment: Data extraction and quality assessment: where is it?

Response: Thank you dear reviewer. We cited the supporting files in the methods section. The results of quality assessment for observation studies were presented in **Table A in** **S1 Table**, whilst the quality assessment result of clinical trial studies was presented in **Table B in S1 Table**. The data extraction results were presented from **Table A to C in S2 Table** in the results section.

Comment: The following data items were extracted: 1) primary author and year of publication; 2) study design; 3) diagnosis confirmation method; 4) sample size; 5) clinical phenotype of CL (LCL, MCL and DCL); 6) treatment detail (route, dose, dosage and treatment type); 7) characteristics of participants; and 8) treatment outcomes. Where is your data that contains the above variables????

Response: Thank you dear reviewer. We cited the supporting file in the methods section and the results that contain data related to the above data items were presented in the results section **from Table A to C in S2 Table**.

**Results**

Comment: Characteristics and quality of included studies. It better to replace by Description of included studies and Characteristics of the included studies subtitles and state accordingly

Response: Thank you dear reviewer for your suggestion and valuable comment. We revised as per your comments. In addition to your suggestion, we added the subsection “search results” in the results section. The changes can be seen in the results section of the revised manuscript.

Comment: Fig. 1 Flow chart of studies’ search and retrieval process should be presented in the main document

Response: Thank dear reviewer. We uploaded the figures separately because of the journal recommendation. The PLOS ONE authors guideline recommended that Figures should be uploaded separately and tables should be uploaded in the main document.

Comment: In Fig.1, report sought retrieval (n=53) and of them 33 studies excluded =when we sum up the number of studies excluded are 19, 19 vs 33????

Response: Thank you dear reviewer for the highest level of curiosity. It was an editorial problem. The 14 studies excluded because responses to therapy were not reported were hidden during figure editorial process. So, we made revision to Figure 1 as per the comment.

Comment: Five studies included from a reference list of articles excluded with reason. Why do you include those studies manually at the beginning in parallel with electronic searching engines

Response: Thank you dear reviewer. Your comments are highly valued. Five articles were included through manual searching of the reference list of excluded articles retrieved. These studies were retrieved from manual searching of the reference list of articles, but we were unable to put parallelly at the beginning because of the spacing to draw the figure.

Comment: Where is your Subgroup analysis result

Response: Thank you dear reviewer. The results of the subgroup analysis were uploaded in Figure as separate TIF file. The Figures 4, 5 and 6 showed the subgroup analysis results.

Comment: Clearly indicate trim and fill analysis for publication bias

Response: Thank you dear reviewer once again for your curiosity. The trim and fill analysis were not performed in our case. Despite the asymmetric funnel plot, the Egger’s regression p-value was 0.329, this implies no publication bias so we did not perform trim and fill analysis.

Comment: What models you used to assess heterogeneity

Response: Thank so much. The I-square test was done to assess the heterogeneity. Random effects model was used to pool the proportion of cure in our study that gave the I-squared statistics that we used to evaluate heterogeneity between studies.

Comment: Tables and figures should be presented in the result section of the main document

Response: Thank dear reviewer. Because of the rules of journal guideline, we were unable to present the figures in the main document. Additionally, the tables we used to present the data items was large to fit in single page border of the Microsoft word, so we included the main results table as supporting file in Tables A to C in S2 Table. All the data items and variables are presented in these supporting file tables.

Comment: Show your Subgroup and sensitivity analysis result in the main document

Response: Thank you dear reviewer once again. The sensitivity analysis and subgroup analysis figure were uploaded separately as per the authors guideline recommendation to submit figures. The sub-group analysis result figures were uploaded as Figure Figures 4, 5 and 6 while Figure 7 showed sensitivity analysis result. We did not include the figures in the main document because of the journal recommendation that the figures should be uploaded separately based on their order as cited in the main document.

**Discussion**

Comment: Compare your finding with another systemic studies and or guidelines. If not available you can compare with studies with large sample size

Response: Thank you dear reviewer. The references used for comparison in the discussion section were systematic reviews and meta-analysis.

Comment: Please discus variables used in subgroup analysis like year…

Response: Thank you reviewer for your comment. We included discussion on the subgroup analysis based on type of treatment and clinical phenotype of CL. We did not perform subgroup analysis by year, so we did not conduct discussion based on year.

Comment: Discus on Predictors of outcome

Response: Thank you dear reviewer. We revised as per the comment and the revision can be seen in track changes in the discussion section.

**References**

Comment: Write your references following standard Vancouver referencing style

Response: Thank you dear reviewer we revised as per the comments, and the changes can be seen

**Comment: In general**: the title itself done previously, I found a lot of errors particularly in the methodology section, topographical errors and also I did not found new compared to the published.

Response 1: Thank you dear reviewer for your scholarly comment. The authors revised the methodology section in detail and the changes made can be seen in track changes in the methodology section of this manuscript.

Response 2: Thank you dear reviewer. The typological errors are copy edited by the University of Gondar editorial center, and I contacted my colleague Mr. Yallew Aklog who is PhD fellow in English language at Bahir Dar University Ethiopia to review the grammar and copy edited the typological errors.

Response 3: Thank you dear reviewer. The authors of this study are grateful for your valid comments, but our study generated the following new and additional evidence.

1. We estimated the pooled proportion of cure based on the meta-analysis of proportions of cure reported from seven studies, this might be taken as additional evidence. However, the previous article did not report the pooled proportion of cure.
2. The secondary outcomes of our systematic review and meta-analysis also revealed the pooled proportions of unfavorable outcomes, such as treatment failure, partial response, dropout and unresponsiveness. Nonetheless, the previous study did not pool evidences on these outcomes. We felt that these reports are also additional evidence to the scientific community to anticipate the proportions of unfavorable treatment outcomes while treating CL due to L. aethiopica.
3. Unlike the previous narrative review, the pooled proportion of cure was reported based on the clinical phenotype of CL and type of treatment. We felt that this is also additional evidence to the existing body of knowledge.
4. After systematic review done in 2016 eight studies published. We included these 8 new studies in our systematic review and meta-analysis that gave us the chance to pool the favorable and unfavorable outcomes of *L. aethiopica* to anti-leishmaniasis therapy, but these reports were not performed in the previous review because the studies were not enough to generate pooled effect size.
5. It has been seven years since the previous systematic review has been published, so our study generated more recent evidence. Additionally, I consulted a senior Biostatician who is an Associate Professor at University of Gondar on the possibility of doing systematic review and meta-analysis in the presence of published systematic review. He affirmed that new systematic review and meta-analysis required after five years to generate additional knowledge, provide new studies are added.

**Point by point response to Reviewer #2 comments**

**General comments to the Author by reviewer #2**

Comment 1. Is the manuscript technically sound, and do the data support the conclusions?

The manuscript must describe a technically sound piece of scientific research with data that supports the conclusions. Experiments must have been conducted rigorously, with appropriate controls, replication, and sample sizes. The conclusions must be drawn appropriately based on the data presented.

Reviewer #2: Yes

Response: Thank you dear reviewer for your encouraging comment.

Comment 2. Has the statistical analysis been performed appropriately and rigorously?

Reviewer #2: Yes

Response: Thank you dear reviewer for your encouraging comment

Comment 3. Have the authors made all data underlying the findings in their manuscript fully available?

The PLOS Data policy requires authors to make all data underlying the findings described in their manuscript fully available without restriction, with rare exception (please refer to the Data Availability Statement in the manuscript PDF file). The data should be provided as part of the manuscript or its supporting information, or deposited to a public repository. For example, in addition to summary statistics, the data points behind means, medians and variance measures should be available. If there are restrictions on publicly sharing data—e.g. participant privacy or use of data from a third party—those must be specified.

Reviewer #2: Yes

Response: Thank you dear reviewer for your encouraging comment

Comment 4. Is the manuscript presented in an intelligible fashion and written in standard English?

PLOS ONE does not copyedit accepted manuscripts, so the language in submitted articles must be clear, correct, and unambiguous. Any typographical or grammatical errors should be corrected at revision, so please note any specific errors here.

Reviewer #2: Yes

Response: Thank you dear reviewer for your encouraging comment

**Specific review comments to the author by reviewer #2**

Comment: I have got the manuscript written well. It brings the important agenda to the board, the treatment outcomes of CL.

Response: Thank you dear reviewer. The authors are grateful for your encouraging comment.

Comment 1. I include the review/publication period in the abstract and method section. E.g. in the review, articles published between 2009-2022 were included

Response: Thank you dear reviewer. This systematic review and meta-analysis did not include restriction by year of publication. This was clearly indicated in the methods section under the eligibility of studies sub-section.

Comment 2. Geographical location of the study. Ethiopia? East Africa? Limit its boundary. 18 studies are from Ethiopia, 3 from migrants and 1 in Kenya. This is good to infer the study findings.

Response: Thank you dear reviewer. Yes, though almost all CL cases (99.9%) in Ethiopia are die to L. aethiopica, case due to this unique species were reported in Mount Elogan area of Kenya, and imported cases with migrants in Israel, Belgium and Germany. So, we did not restrict the studies by geographical location to include studies done on CL due to L. aethiopica in other settings.

Comment 3. Abbreviation NOS (Non-observational studies?) not clear. Clarify it (page 6)

Response: Thank you dear reviewer. An abbreviation ‘NOS’ refers to ‘Newcastle Ottawa Scale’, and the definition of this abbreviation has been included in the methods section as well as list of abbreviations section of the manuscript.

Comment 4. Your suggestion and recommendation regarding local therapy for CL due to L. aethiopica.

Response: Thank you dear reviewer. Yes, the findings showed that local therapy had better cure rate, but the use of local therapy was limited to LCL cases. However, combination therapy showed higher cure rate across all clinical phenotypes (LCL, MCL and DCL).

1.Schulzke S. Assessing and Exploring Heterogeneity. Principles and Practice of Systematic Reviews and Meta-Analysis: Springer; 2021. p. 33-41.

2.Patole S. Systematic reviews, meta-analysis, and evidence-based medicine. Principles and practice of systematic reviews and meta-analysis: Springer; 2021. p. 1-10.

3.Haidich A-B. Meta-analysis in medical research. Hippokratia. 2010;14(Suppl 1):29.
