# Supplementary material for: Treatment outcomes of cutaneous leishmaniasis due to Leishmania aethiopica: A systematic review and meta-analysis
Source: PLoS One. 2023 Nov 2;18(11):e0293529. doi: 10.1371/journal.pone.0293529 (PMC10621858; doi:10.1371/journal.pone.0293529)
Supplement: Attachment — Submitted filename: Response to Reviewers.docx [file pone.0293529.s011.docx]

**Point-by-point response letter**

Dears Academic Editor (Dr. Balew Arega Negatie, MD, MSc), Reviewer #2, Reviewer #3 and Reviewer #4,

Thank you for your constructive comments that we missed in the first round of revision. The authors appreciate your constructive editorial comments that could help to improve the readability and impact of our work. The order of citations in the supporting files S1 Table and S2 Table are updated, and we also uploaded the revised Figure 5.

In this point-by-point response here below, we highlighted the response in light black color below the respective comment (s) by the editor and the reviewers. First, we have presented the comments and the authors’ response to the editor's comments. Then, we presented our responses to the comments by Reviewer #2, Reviewer #3 and Reviewer #4, respectively.

**Point-by-point response to editors’ comment**

**Additional Editor Comments:**

Comment 1. This comment in the previous version must be addressed “In Figure 5, there is only one study in the subgroup analysis (Seife T/2018). However, there must be at least two studies in each subgroup. The findings of this analysis skewed your conclusion and recommendation. This must be corrected and revise your conclusion accordingly”. You can remove this study from the sub-group MA and do it for others. You can narrate about this study. Dear author, the subgroup meta-analysis is just meta-analysis, so no possibility of doing an MA for a single study.

Response: Thank you, dear editor. Your comments are highly valuable. We did a meta-analysis after removing the study by Seife et al. and uploaded the revised figure as Fig 5. We also revised the conclusion accordingly. We include all the revisions in the revised manuscript in track changes. The revised subgroup analysis result is included in the revised track change version of the manuscript on page 11, lines 272-280, in the results section. We revised the conclusion in track change in the abstract sections and the conclusions sections accordingly.

Comment 2. The manuscript has good progress, but it needs extensive language editing. I tried to highlight the most important ones.

Response: Thank you indeed dear editor for editing our work line by line. We forward our heartful thanks for editing our work to make it readable. We have extensively edited the manuscript, and proofread the manuscript. The edits can be seen in track changes from the first page to the end.

**Point-by-point response to Reviewer #2**

Comment 1: All comments have been addressed

Response: Dear reviewer # 2, thank you for your review. We forward our sincerest thanks for your time spent reviewing our work and responding to that we have addressed your comments.

**Point-by-point response to Reviewer #3**

**General comment**

Comment 1: The authors performed a systematic review and meta-analyzed based on literature searches together evidence on treatment outcome of CL caused by L. aethiopica. They concluded that combination therapy was the better option to increase the cure rate. However, minor revisions are needed before being considered for publication.

Response: Thank you dear reviewer for your comment. The manuscript has been revised intensively as per your comments, and the changes made can be seen in the track change in the revised version of the manuscript starting from the first page to the end.

**Specific comment**

Comment 1:- L3 (Introduction), correct L. tropica writing properly.

Response: Thank you very much dear reviewer. We have revised as per your comment and the changes included in the track change in line 42 in the introduction section. We have used the italic style because L. tropica is the name of the species. As per the PLOS ONE recommendation, we wrote species name in italic style.

Comment 2: - L4 (Introduction), correct L. major writing properly.

Response: Response: Thank you very much dear reviewer. We have revised as per your comment and the changes included in the track change on page 2, line 42, in the introduction section. We have used the italic style because L. tropica is the name of the species. As per the PLOS ONE recommendation, we wrote the species name in italic style.

Comment 4: - The authors should present a brief introductory paragraph about the life cycle of the parasite describing the primary vectors and the principal reservoir hosts and the abundance of the disease in the area.

Response: Thank you very much, dear reviewer. We included a brief statement about the vectors and reservoirs of *L. aethiopica* in the introduction section. We included the statement: Two hyrax species (Heterohyrax brucei and Procavia capensis), and sandfly (Phlebotomus longipes and Ph. Pedifer) species are the known reservoirs and vectors of L. aethiopica. You can check the changes in the revised version of the manuscript in track changes on page 2, lines 44-46 in the introduction section.

Comment 4: - In the discussion section, the authors should describe some of the limitations of the analyzed studies, including the lack of clinical trials and analytical studies, such as cohort studies and so on.

Response: Thank you indeed, dear reviewer. We have included the limitations of this study and the reviewed studies in the last paragraph of the discussion section of the revised manuscript from on page 15, lines 377-386.

Comment 5: - A moderate revision of the text is required.

Response: Thank you again for the comment. We have extensively edited the manuscript and proofread it. Dear reviewer, the edits can be seen in track change throughout the entire manuscript from the first page to the end.

**Point-by-point response to Reviewer # 4**

Comment 1: Since you included all CL cases who are treated using both single and combined forms of the treatment. So better to replace it with "treatment outcome".

Response: Thank you reviewer for your comment. We revised the as per the comment, and we include this revision in the revised manuscript in the track change on page 2, line 38, in the abstract section.

Comment 2: Did you ever check papers using other search engines/strategies like HINARI, CINHAL.....

Response: Thank you so much, dear reviewer. We searched three databases (PubMed, Scopus and ScienceDirect), so we addressed the minimum requirement of three database searches. In addition, CINHAL is not accessible in our country because it requires a subscription. Also, we searched Google Scholar and the reference list of articles to increase the inclusion of potential pocket studies. Furthermore, the two databases, Scopus and PubMed are widely indexed databases in health research, so we believe we did not miss relevant studies. Moreover, articles in HINARI are accessible through PubMed and articles in Embase is accessible in Scopus.

Comment 3: Sure, you can use this risk of bias assessment tool. But why do not like to use the QOUREM tool for these RCTs?

Response: Thank you, dear reviewer. We chose the revised risk of bias assessment (RoB 2) tool because it is easy to use. The RoB 2 can assess the risk of bias based on five important domains: (1) risk of bias arising from the randomization process, (2) risk of bias due to adherence or assignment to the intended intervention, (3) risk of bias due to missing outcome data, (4) risk of bias in the measurement of outcome, (5) risk of bias in the selection of reported results. The tool is also applicable to all types of RCTs. Additionally, the RoB2 classifies studies into high, medium, and low risk based on the assessors' scores for these five domains. However, we acknowledge the availability of other assessment tools, as you stated clearly.

Comment 4: Better to change the phrase Clinical cure status and the like

Response: Dear reviewer, thank you for the comment. We revised as per your comment and replaced the wording positive outcome with Favourable outcomes. We use the word favourable instead of clinical cure because in the studies the authors report as “cure, improved, negative/decrease in parasite density, good response, decrease in the size of the lesion, and resolution of the lesion without scar”. Hence, we use favourable outcomes instead of positive outcomes. The change can be seen in the revised version of the manuscript in track change, starting from page 7 line 179, in the results section.

Comment 5: What do you feel if someone says negative outcome is: something comes secondary to a certain agent, adverse event and the like? So better to replace it with another better descriptive word, please. Like "Nil, poor, ...... responsive ".

Response: Thank you indeed, dear reviewer. We have revised as per your comment and replaced the word negative outcomes with Unfavourable outcomes. The changes can be seen in track change in the revised version, starting from page 9, line 226, in the results section. The word unfavourable was deemed more descriptive for the authors because the studies we reviewed report a range of outcomes such as recurrence, resistance, treatment extension, partial response, relapse, dropout, and unresponsiveness, progression to a more severe stage and toxicity. So, we chose the word unfavourable which we believe best described these outcomes.

Comment 6: Include the limitation of the study.

Response: Thank you, dear reviewer. We have included the limitation of this study and the studies we included in this review in the last paragraph of the discussion section, page 15, from lines 377-386. The response to these comments is included in the track change in the revised version of the manuscript.
