# Supplementary material for: Treatment outcomes of cutaneous leishmaniasis due to Leishmania aethiopica: A systematic review and meta-analysis
Source: PLoS One. 2023 Nov 2;18(11):e0293529. doi: 10.1371/journal.pone.0293529 (PMC10621858; doi:10.1371/journal.pone.0293529)
Supplement: Attachment — Submitted filename: Response to Reviewers.docx [file pone.0293529.s012.docx]

**Point-by-point response letter**

Dears Academic Editor (Dr. Balew Arega Negatie, MD, MSc) and Reviewer #3 (Professor Iraj Sharifi, PhD, Managing Director at Kerman University of Medical Sciences, Iran)

Thank you for your constructive comments that we missed in the second round of the revision. The authors forward heartfelt gratitude for your scholarly insights and comments to increase the readability of our manuscript.

In this point by point response letter, first, we respond to the query on journal requirements about the reference list in the manuscript. Followed by point-by-point response for the comments by Reviewer #3. In this point-by-point response here below, we highlighted the author response in light black color below the respective comment (s) by the reviewer.

**Point by point response to the Journal Requirements query**

Comment: Please review your reference list to ensure that it is complete and correct. If you have cited papers that have been retracted, please include the rationale for doing so in the manuscript text, or remove these references and replace them with relevant current references. Any changes to the reference list should be mentioned in the rebuttal letter that accompanies your revised manuscript. If you need to cite a retracted article, indicate the article’s retracted status in the References list and also include a citation and full reference for the retraction notice.

Author response: Thank you dear editor. We reviewed the reference list are complete and correct, and ensured that no reference (s) cited were retracted. In addition, all the references are based on the Vancouver style as per the PLOS ONE requirement.

**Point by point response to reviewer comments**

**Comments to the Author**

1. If the authors have adequately addressed your comments raised in a previous round of review and you feel that this manuscript is now acceptable for publication, you may indicate that here to bypass the “Comments to the Author” section, enter your conflict of interest statement in the “Confidential to Editor” section, and submit your "Accept" recommendation.

Reviewer #3: All comments have been addressed

Author response: Thank you, dear reviewer, for replying that we have addressed your comments.

2. Is the manuscript technically sound, and do the data support the conclusions?

Reviewer #3: Yes

Author response: Thank you, dear reviewer, for your encouraging response and valuing our efforts.

3. Has the statistical analysis been performed appropriately and rigorously?

Reviewer #3: Yes

Author response: Dear reviewer, thank you again for your encouraging response and valuing our efforts of using rigorous methodology.

4. Have the authors made all data underlying the findings in their manuscript fully available?

Reviewer #3: Yes

Author response: Dear reviewer, thank you indeed.

5. Is the manuscript presented in an intelligible fashion and written in standard English?

   Reviewer #3: Yes

Author response: Dear reviewer, thank you again for your encouraging response and valuing our efforts of reviewing to meet the standard English language requirements

6. Review Comments to the Author

Review comment 1: L45, the name of the species should be written in small font (e.g., Ph. pedifer).

Author response: Thank you, dear reviewer, for reading our work line by line. We revised the species name as per your comment and the change can be seen on line 45, page 2, in the tracked change version of the manuscript.

Review comment 2: L113, Leishmania aethiopica should be italicized.

Author response: Thank you, dear reviewer, for reading our work line by line. We revised the species name as per your comment and the change can be seen on line 113, page 5, in the tracked change version of the manuscript.

Review comment 3: L355, Leishmania should be written correctly (the “L” should be capitalized).

Author response: Thank you, dear reviewer, for reading our work line by line. We revised the species name as per your comment and the change can be seen on line 355, page 14, in the tracked change version of the manuscript.
